# Multi-omics analysis delineates the distinct functions of sub-cellular acetyl-CoA pools in *Toxoplasma gondii*

**DOI:** 10.1186/s12915-020-00791-7

**Published:** 2020-06-16

**Authors:** Joachim Kloehn, Rebecca D. Oppenheim, Ghizal Siddiqui, Pieter-Jan De Bock, Sunil Kumar Dogga, Yohann Coute, Mohamed-Ali Hakimi, Darren J. Creek, Dominique Soldati-Favre

**Affiliations:** 1 Department of Microbiology and Molecular Medicine, CMU, University of Geneva, Rue Michel-Servet 1, 1211 Geneva, Switzerland; 2 Drug Delivery, Disposition and Dynamics, Monash Institute of Pharmaceutical Sciences, Monash University, Parkville campus, Parkville, VIC 3052 Australia; 3 University Grenoble Alpes, CEA, INSERM, IRIG, BGE, F-38000 Grenoble, France; 4 Epigenetic and Parasites Team, UMR5163/LAPM, Domaine de la Merci, Jean Roget Institute, 38700 La Tronche, France

**Keywords:** *Toxoplasma gondii*, Acetyl-CoA, Branched-chain α-keto acid dehydrogenase-complex (BCKDH), ATP citrate lyase (ACL), Acetyl-CoA synthetase (ACS), Acetylome, Multi-omics, Metabolism, Phosphoenolpyruvate carboxykinase (PEPCK), Formate/nitrite transporter (FNT)

## Abstract

**Background:**

Acetyl-CoA is a key molecule in all organisms, implicated in several metabolic pathways as well as in transcriptional regulation and post-translational modification. The human pathogen *Toxoplasma gondii* possesses at least four enzymes which generate acetyl-CoA in the nucleo-cytosol (acetyl-CoA synthetase (ACS); ATP citrate lyase (ACL)), mitochondrion (branched-chain α-keto acid dehydrogenase-complex (BCKDH)) and apicoplast (pyruvate dehydrogenase complex (PDH)). Given the diverse functions of acetyl-CoA, we know very little about the role of sub-cellular acetyl-CoA pools in parasite physiology.

**Results:**

To assess the importance and functions of sub-cellular acetyl-CoA-pools, we measured the acetylome, transcriptome, proteome and metabolome of parasites lacking ACL/ACS or BCKDH. We demonstrate that ACL/ACS constitute a synthetic lethal pair. Loss of both enzymes causes a halt in fatty acid elongation, hypo-acetylation of nucleo-cytosolic and secretory proteins and broad changes in gene expression. In contrast, loss of BCKDH results in an altered TCA cycle, hypo-acetylation of mitochondrial proteins and few specific changes in gene expression. We provide evidence that changes in the acetylome, transcriptome and proteome of cells lacking BCKDH enable the metabolic adaptations and thus the survival of these parasites.

**Conclusions:**

Using multi-omics and molecular tools, we obtain a global and integrative picture of the role of distinct acetyl-CoA pools in *T. gondii* physiology. Cytosolic acetyl-CoA is essential and is required for the synthesis of parasite-specific fatty acids. In contrast, loss of mitochondrial acetyl-CoA can be compensated for through metabolic adaptations implemented at the transcriptional, translational and post-translational level.

## Background

The phylum Apicomplexa groups a range of obligate intracellular parasites including *Plasmodium* spp., *Cryptosporidium parvum* and *Toxoplasma gondii*, the causative agents of malaria, gastrointestinal disease and toxoplasmosis, respectively. The metabolism of these pathogens is an area of intense research, aiming to identify new drug targets and develop inhibitors with new chemotypes to overcome the limitations of current drugs, such as high cost, toxicity and emerging resistance [1, 2].

**Fig. 1:**
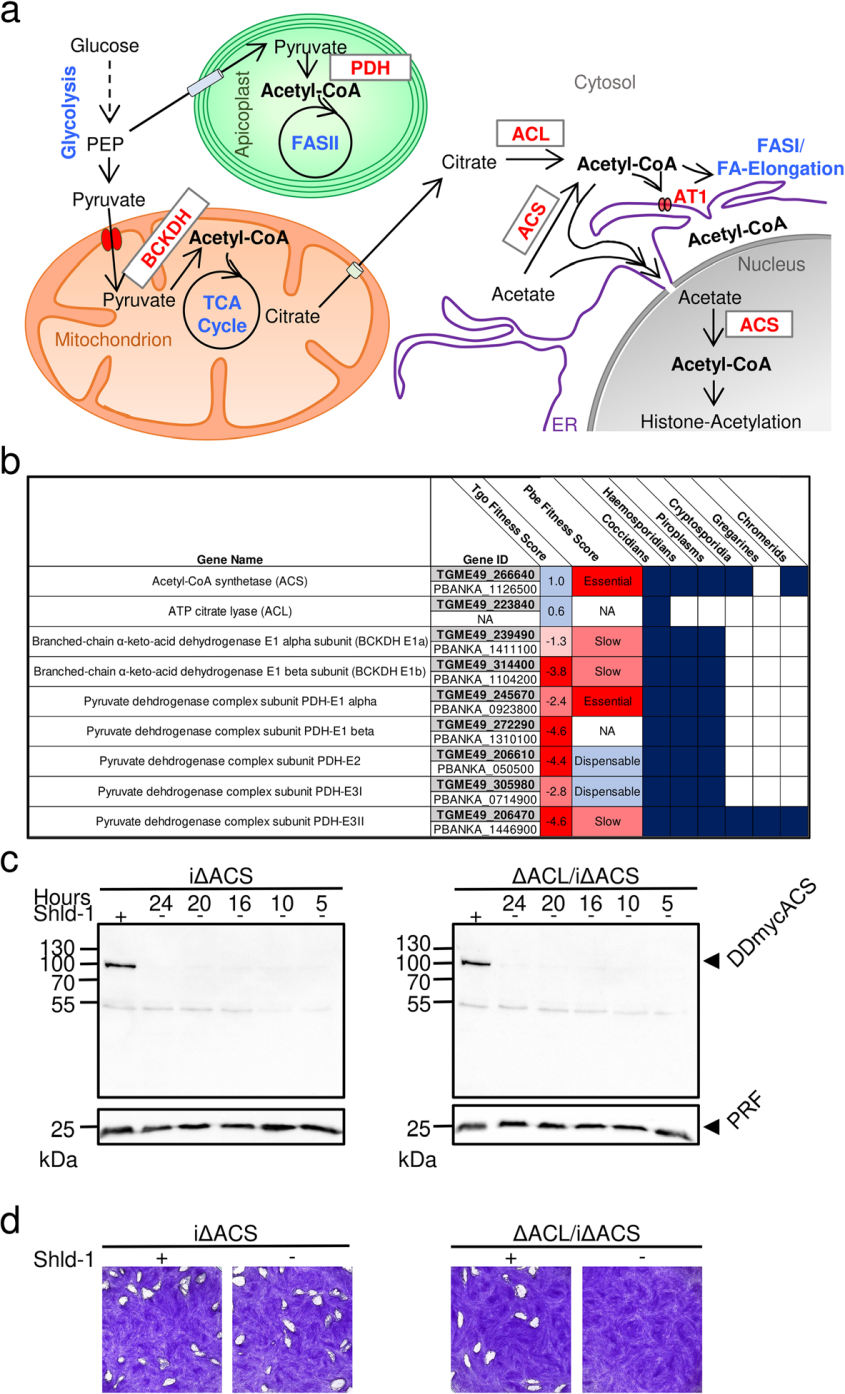
ACS and ACL are essential to produce acetyl-CoA in the cytosol and nucleus. **a** Schematic representation of the metabolic pathways in *T. gondii* for acetyl-CoA production and transport into the cellular compartments where it is required: the apicoplast, mitochondrion, cytosol, nucleus and the endoplasmic reticulum (ER). Metabolic pathways are highlighted in blue and enzymes in red, and metabolites are depicted in black. BCKDH, branched-chain α-keto acid dehydrogenase-complex; PDC, pyruvate dehydrogenase complex; FA, fatty acid; FASII, type II FA synthase, ACL, ATP citrate lyase; ACS, acetyl-CoA synthetase; AT1, acetyl-CoA transporter; ER, endoplasmic reticulum; TCA, tricarboxylic acid. **b** Table highlighting the essentiality [10, 11] of acetyl-CoA-generating enzymes in *T. gondii* (Tgo) and *Plasmodium berghei* (Pbe) and their conservation across different apicomplexans. **c** Immuno-blot of total protein lysates from iΔACS (left panel) and ΔACL/iΔACS parasites (right panel) for which Shield-1 (Shld-1) was removed at several time points prior to egress to test protein regulation. Western blots were probed using α-myc antibody to detect the myc-tag of DD-ACS, and α-profilin (PRF) was used as a loading control. **d** Plaque assays were performed by inoculating human foreskin fibroblast (HFF) monolayers with either iΔACS, or ΔACL/iΔACS parasite strains and left to grow in the presence (+) or absence (−) of Shld-1 over a period of 7 days. Plaques were revealed by crystal violet staining of infected HFF monolayers

*T. gondii* harbours several metabolically active sub-cellular compartments including the cytosol, nucleus, endoplasmic reticulum (ER), Golgi apparatus, mitochondrion and apicoplast, a relict plastid-like organelle derived from secondary endosymbiosis and possibly peroxisomes, which may form in the oocyst/sporozoite stage [3–5]. Acetyl-CoA is a hub metabolite with distinct and crucial functions in each of these compartments, involved in several anabolic and catabolic pathways and crucial for the acetylation of histones as well as non-histone proteins [6–9]. Due to its amphiphilic nature and high molecular weight, acetyl-CoA cannot freely cross the membranes and must be produced within, or actively transported into, the compartments which rely on acetyl-CoA (Fig. 1a) [9]. In *T. gondii*, the pyruvate dehydrogenase complex (PDH) converts pyruvate to acetyl-CoA in the apicoplast [12]. In the mitochondrion, the branched-chain α-keto acid dehydrogenase-complex (BCKDH) replaces the function of PDH to generate acetyl-CoA from pyruvate [13]. Two complementary routes generate acetyl-CoA in the cytosol and nucleus: the acetyl-CoA synthetase (ACS) produces acetyl-CoA from acetate, and the ATP citrate lyase (ACL) converts citrate to acetyl-CoA [14]. A putative acetyl-CoA transporter (AT-1) likely enables the import of cytosolic acetyl-CoA into the ER [14, 15]. If, and during which life cycle stages, acetyl-CoA is generated by β-oxidation in *T. gondii* is unclear [5].

The high negative scores in a recent fitness screen of *T. gondii* metabolic genes indicate fitness-conferring roles of PDH and BCKDH (Fig. 1b) [16]. In contrast, the low positive scores of ACS and ACL indicate dispensability, consistent with a previous study which demonstrated that ACS and ACL are synthetic lethal [14]. Apart from coccidians, other apicomplexans, including the malaria parasites, lack ACL and thus rely solely on ACS to generate acetyl-CoA in the nucleo-cytosol (Fig. 1b). Consequently, ACS is predicted to be essential in *Plasmodium* as indicated by genome-wide fitness screens and is considered as a promising drug target [10, 17, 18].

While previous studies focused on determining the extent of protein acetylation in apicomplexans and identified differences between parasite stages or strains [19–22], little is known about the specific roles of distinct acetyl-CoA pools and how these impact parasite physiology. Here, we combined multi-omics analysis with molecular tools to reveal the diverse functions of acetyl-CoA in *T. gondii*.

## Results

### Loss of nucleo-cytosolic acetyl-CoA production is detrimental and causes morphological defects in *T. gondii*

We have previously postulated that both ACL and ACS contribute to the generation of acetyl-CoA in the cytosol and nucleus and constitute a synthetic lethal pair in *T. gondii* [14]. To elucidate the role of ACL and ACS, we generated an inducible conditional knock-down of ACS in RH parasites (iΔACS) as well as in parasites in which *acl* was deleted by double-homologous recombination (ΔACL/iΔACS). For the conditional knock-down, a destabilisation domain (DD) was fused to a myc-tag at the N-terminus of ACS in the endogenous *acs* gene locus by CRISPR/Cas9-mediated genome editing (Additional file 1: Figure S1a). The DD allows for rapid proteasome degradation in the absence of the protective ligand Shield-1 (Shld-1) [23]. Integration of the DDmyc in the *acs* locus and replacement of the *acl* open reading frame (ORF) with the hypoxanthine-xanthine-guanine phosphoribosyl transferase (HXGPRT) resistance cassette (Additional file 1: Figure S1a, b) were validated by PCR analysis of genomic DNA (Additional file 1: Figure S1c). Expression and effective regulation of the DDmyc-ACS fusion protein in iΔACS and ΔACL/iΔACS parasites were confirmed by western blot (Fig. 1c).

**Fig. 2:**
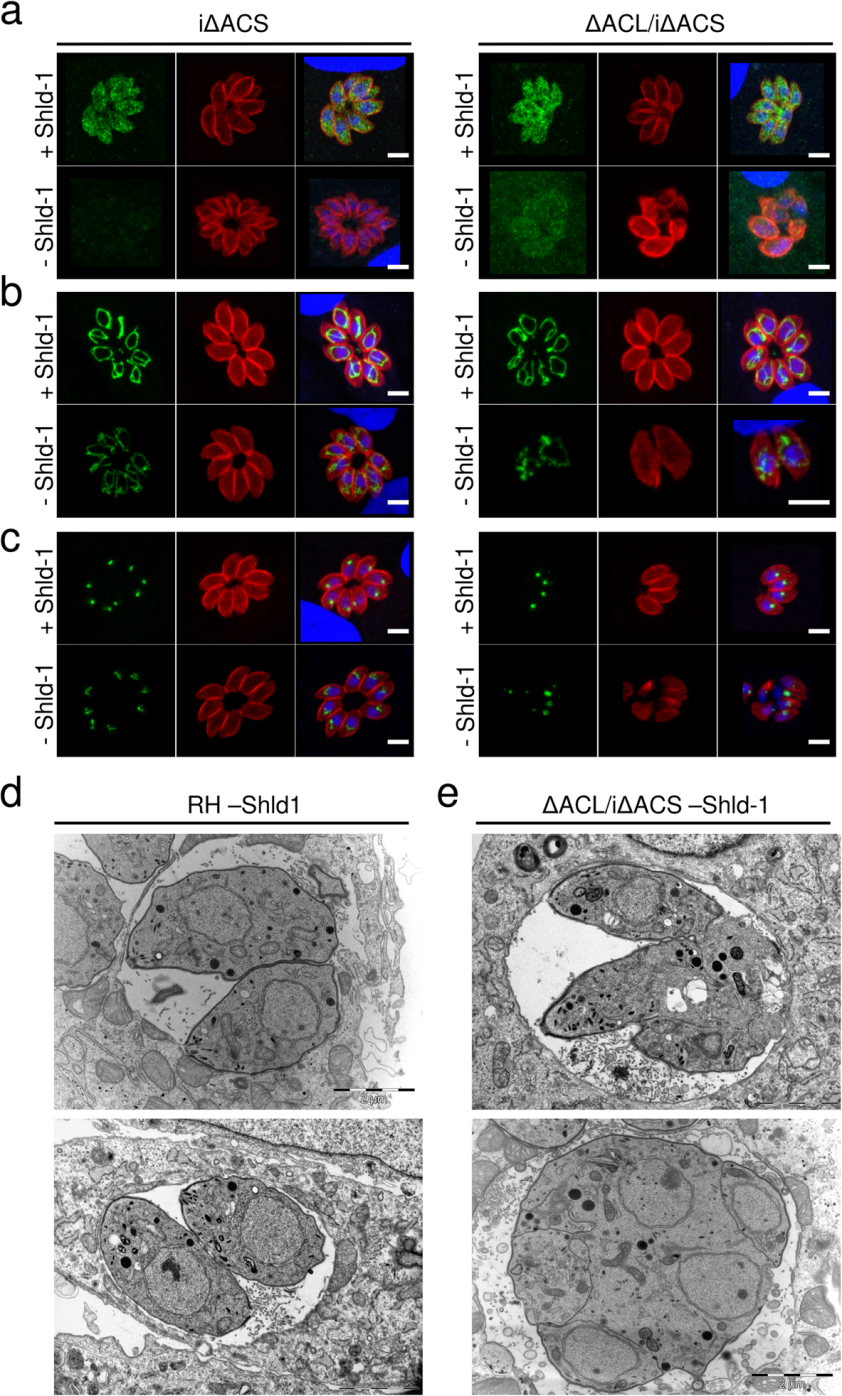
Loss of ACS and ACL is associated with amorphic cells and loss of organelle integrity. Immunofluorescence assays (IFAs) of intracellular iΔACS or ΔACL/iΔACS parasites grown in the presence (+) or absence (−) of Shield-1 (Shld-1) for 24 h (**a**–**c**). IFAs were fixed and stained with α-gliding-associated protein 45 (α-GAP45, red) to show pellicles of the parasites and 4′,6 diamidin-2-phenylindol (DAPI, blue) to stain the nuclei and either, α-myc (green) to detect ACS (**a**) or with the monoclonal antibody 5F4 (α-F1B ATPase, green) marking the mitochondrion (**b**) or α-apicoplast-associated thioredoxin family protein 1 (α-Atrx1, green) staining the apicoplast (**c**) (scale bars, 5 μm). Electron micrographs of intracellular RH (**d**) or ΔACL/iΔACS (**e**) grown in the absence of Shld-1 for 24 h (scale bars, 2 μm). ACL, ATP citrate lyase; ACS, acetyl-CoA synthetase

In plaque assays, which assess several lytic cycles, iΔACS or ΔACL parasites showed no defect compared to RH parasites grown in human foreskin fibroblasts (HFFs) over 1 week (Fig. 1d). In contrast, ΔACL/iΔACS parasites failed to form plaques after 7 days in the absence of Shld-1 (Fig. 1d), confirming our previous prediction of synthetic lethality [14]. Immunofluorescence assays (IFAs) revealed that iΔACS and ΔACL parasites were morphologically normal, while ΔACL/iΔACS parasites presented a severe impairment in cell division with a loss of pellicle integrity as seen by clear alteration of staining of a pellicle marker, the gliding-associated protein 45 (GAP45) at 24 h after Shld-1 removal (Fig. 2a). Additionally, staining of the mitochondrion and apicoplast appeared diffuse in ΔACL/iΔACS parasites, suggesting a loss of integrity of both organelles (Fig. 2b, c). Similarly, electron microscopy examination of ΔACL/iΔACS highlighted extreme morphological defects 24 h after Shld-1 removal (Fig. 2d, e). Some dividing ΔACL/iΔACS parasites presented loss of the basal structure (Fig. 2e, top panel), or were entirely amorphic, with the contents of multiple fused parasites enclosed by a joined plasma membrane (Fig. 2e, bottom panel). Altogether, these results support our previous observation that ACL and ACS constitute a synthetic lethal pair, depletion of which causes arrest of parasite growth.

### Lack of ACL/ACS alters the *T. gondii* metabolome including disruption of FA elongation

**Fig. 3:**
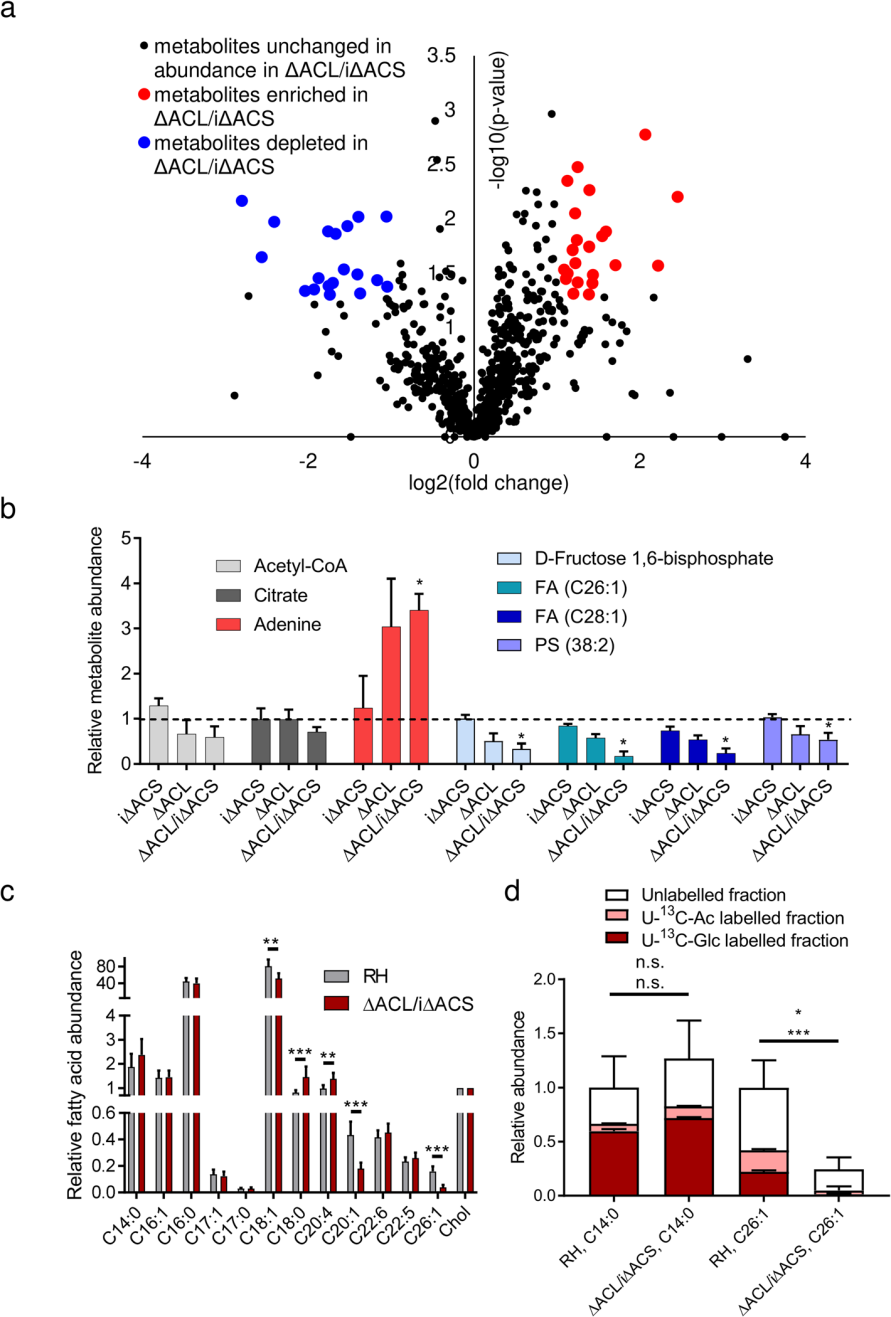
Loss of ACS and ACL causes changes in the *T. gondii* metabolome including a halt in FA elongation. **a** Volcano plot highlighting changes in metabolite levels of ΔACL/iΔACS compared to RH parasites. Unchanged metabolites are displayed in black, while significantly increased or decreased metabolites (≥ 2-fold change, *p* < 0.05) in ΔACL/iΔACS are displayed in red and blue, respectively. Statistically significant differences were assessed using a *t*-test comparing triplicates of ΔACL/iΔACS parasites to triplicates of RH in the absence of Shield-1. **b** Relative abundance of selected metabolites compared to levels in RH-Shield-1 (dashed line), which are not significantly altered (acetyl-CoA, citrate), significantly decreased (d-fructose 1,6 bisphosphate, FAs C26:1 and C28:1, phosphatidylserine—PS38:2) or significantly increased (adenine) upon loss of ACS and ACL (ΔACL/iΔACS). Error bars represent the standard deviation between replicates (*n* = 3). Statistical significance was assessed using a *t*-test and is indicated (*p < 0.05). **c** GC-MS measurement of FA abundances normalised to cholesterol levels. Error bars represent the standard deviation between replicates (*n* = 6). Statistical significance was assessed by a *t*-test and is indicated (**p < 0.005; ***p < 0.0001). **d** Relative abundance and ^13^C-labelling in myristate (C14:0) and FA C26:1, following incubation in medium containing U-^13^C-glucose or U-^13^C-acetate for 16 h during simultaneous ACS depletion. Error bars represent the standard deviation between replicates. Top error bars represent the standard variation in abundance, and lower error bars represent the standard deviation in labelling between replicates (*n* = 6 for abundance measurement, *n* = 3 for labelling analysis). Statistical significance for abundances as indicated in **c**. Statistical significance was tested using a *t*-test. Differences in C14:0 labelling between RH ΔACL/iΔACS and are non-significant (n.s.). Statistically significant differences in U-^13^C-acetate (*p* < 0.05) and U-^13^C-glucose-labelling of C26:1 between RH and ΔACL/iΔACS are indicated (*p < 0.05; ***p < 0.0001), respectively. ACL, ATP citrate lyase; ACS, acetyl-CoA synthetase; FA, fatty acid; GC-MS, gas chromatography-mass spectrometry; Glc, glucose; Ac, acetate

Analysing ΔACL/iΔACS parasites by IFA and western blot allowed us to conclude that 16 h of Shld-1 removal was sufficient to deplete ACS in the ΔACL/iΔACS strain, while parasites showed no growth defect or morphological abnormalities at this relatively early time point of ACS depletion and remained viable. In the following analyses, iΔACS and ΔACL/iΔACS refer to parasites depleted of ACS by removal of Shld-1 for 16 h. To obtain a global picture of the impact of the loss of nucleo-cytosolic acetyl-CoA on parasite metabolism, we performed untargeted metabolomics using liquid chromatography-mass spectrometry (LC-MS). The full dataset is available in a data repository [24]. Over 850 putative metabolites were detected, and the relative abundance of all metabolites was compared to that of RH parasites (Additional file 2: Table S1). We focused our analysis on putative metabolites which changed more than 2-fold in abundance (*p* value < 0.05) and identified 40 putative metabolites as significantly perturbed, with 19 displaying decreased levels, while 21 were increased in abundance in ΔACL/iΔACS parasites (Fig. 3a, Additional file 2: Table S1). While the loss of nucleo-cytosolic acetyl-CoA affected several metabolic pathways, many clustered into lipid/fatty acyl- and peptide metabolism (Additional file 2: Table S1, Additional file 3: Figure S2a). Enzymes producing or consuming the affected metabolites were almost exclusively predicted to localise to the cytosol or ER (Additional file 2: Table S1). Knock-out of ACL or knock-down of ACS alone had only minor impact with 14 or 1 putative metabolite changing, respectively (Additional file 2: Table S1). However, in many cases, loss of ACL alone led to a modest, non-significant drop in levels of certain metabolites, while the lack of both enzymes (ΔACL/iΔACS) aggravated the phenotype, indicating that the two enzymes have partially redundant functions in metabolism (Fig. 3b, Additional file 2: Table S1). Purine metabolites (adenine, inosine monophosphate (IMP), xanthine) were found increased in parasites lacking ACL and are likely associated with the HXGPRT resistance gene (Additional file 3: Figure S2a). Acetyl-CoA-levels decreased in ΔACL and ΔACL/iΔACS parasites to about 60% compared to RH cells, although the drop was not statistically significant (Fig. 3b). This indicates that the major pools of acetyl-CoA are in the mitochondrion and apicoplast, unaffected by the loss of ACS and ACL, consistent with the dramatic drop in total acetyl-CoA in ΔBCKDH parasites [13].

Major changes were observed in the abundance of monounsaturated very long-chain FAs (FA C26:1, C28:1) by untargeted LC-MS analysis (Fig. 3b), consistent with acetyl-CoA being required for the elongation of FAs on the cytosolic site of the ER [25]. This defect in FA elongation in ΔACL/iΔACS parasites was further scrutinised by semi-targeted profiling of FAs by gas chromatography-MS (GC-MS) (Fig. 3c). Using GC-MS, we found that the relative levels of five detected FAs were significantly altered between RH and ΔACL/iΔACS parasites. While C18:0 and C20:4 were slightly increased in ΔACL/iΔACS parasites, FA C18:1, C20:1 and C26:1 were significantly decreased, with FA C20:1 and C26:1 displaying a dramatic 2-fold and 4-fold drop, respectively, while FA C28:1 was not detected by GC-MS (Fig. 3c). Importantly, most FAs are abundant in the host cells (HFFs) and can be salvaged by *T. gondii*. In particular, C18:0 and C20:4 make up a higher proportion of total FAs in the host compared to *T. gondii* [16]. Instead, C18:1 is of lower relative abundance in the host compared to *T. gondii*, and FAs C20:1, C26:1 and C28:1 are of very low abundance or absent in HFFs [16, 25]. We conclude that *T. gondii* ΔACL/iΔACS compensate for the halt in FA elongation through increased uptake of unsaturated long-chain FAs from the host. The differences in FA abundances in HFFs compared to *T. gondii* [16] lead to the altered FA composition of ΔACL/iΔACS parasites. Additionally, we confirmed the defect in FA elongation by labelling with U-^13^C-glucose or U-^13^C-acetate for 16 h simultaneous to the ACS downregulation followed by GC-MS analysis. ^13^C-labelling in FA C26:1 was significantly decreased in ΔACL/iΔACS parasites confirming the specific loss of FA elongation in these parasites (Fig. 3d, Additional file 3: Figure S2b). In contrast, the abundance as well as ^13^C-labelling in myristate (C14:0) was similar or increased in ΔACL/iΔACS compared to RH parasites, indicating that cells are metabolically active and that de novo FA synthesis in the apicoplast is not affected (Fig. 3d, Additional file 3: Figure S2b). Possibly related to the reduced levels of long and very long monounsaturated FAs, abundance of the lipid phosphatidylserine (PS 38:2) was also found more than 2-fold reduced in ΔACL/iΔACS parasites by LC-MS analysis (Fig. 3b). This may be a consequence of the altered abundance of C18:1 and C20:1, both of which were found to be significantly reduced in ΔACL/iΔACS by semi-targeted GC-MS FA profiling, although the FA composition of the affected PS38:2 was not further scrutinised using tandem MS.

While the synthesis of monounsaturated very long chain FAs depends on acetyl-CoA as a substrate, the drop in levels of other metabolites such as d-fructose-1,6-bisphosphate (F1,6-BP) is unexpected and may result from the altered acetylation status of enzymes in the pathway (Fig. 3b).

Additionally, we performed U-^13^C-glucose and U-^13^C-acetate labelling followed by LC-MS analysis, to compare the utilisation of these metabolites between RH and ΔACL/iΔACS parasites (Additional file 4: Figure S3a). We observed no differences in glucose utilisation when monitoring glycolytic and TCA cycle intermediates. While U-^13^C-glucose labelling resulted in rapid and extensive labelling of glycolytic and TCA cycle intermediates, U-^13^C-acetate resulted in no or very little labelling of central carbon metabolites apart from citrate (Additional file 4: Figure S3b,c). Incubation in U-^13^C-acetate resulted in 50% ^13^C-labelling in the citrate of RH, which was reduced more than 3-fold in iΔACS and ΔACL/iΔACS parasites (Additional file 4: Figure S3c). The lack of/low levels of ^13^C-labelling in other TCA cycle intermediates indicate that the labelling observed in citrate is not in the mitochondrial pool but rather in the cytosolic or a putative apicoplast pool. We propose that a second citrate synthase 2, for which the localisation is yet unknown [26], condenses acetyl-CoA and oxaloacetate (OAA) to form citrate in the cytosol utilising acetyl-CoA generated by ACS which is derived from U-^13^C-acetate. We also observed a significant decrease in labelling from U-^13^C-acetate in some lipid species (Additional file 4: Figure S3d-f) including PS38:2 (Additional file 4: Figure S3f) which was also 2-fold reduced in abundance in ΔACL/iΔACS parasites (Fig. 3b). Overall, our metabolomic analyses demonstrate that lack of ACL/ACS results in several changes in the metabolism, most notably the loss of long and very long monounsaturated FAs, which have previously been demonstrated to be essential for *T. gondii* [25].

### Lack of ACL/ACS or BCKDH results in hypo-acetylation of cytosolic and mitochondrial proteins, respectively

**Fig. 4:**
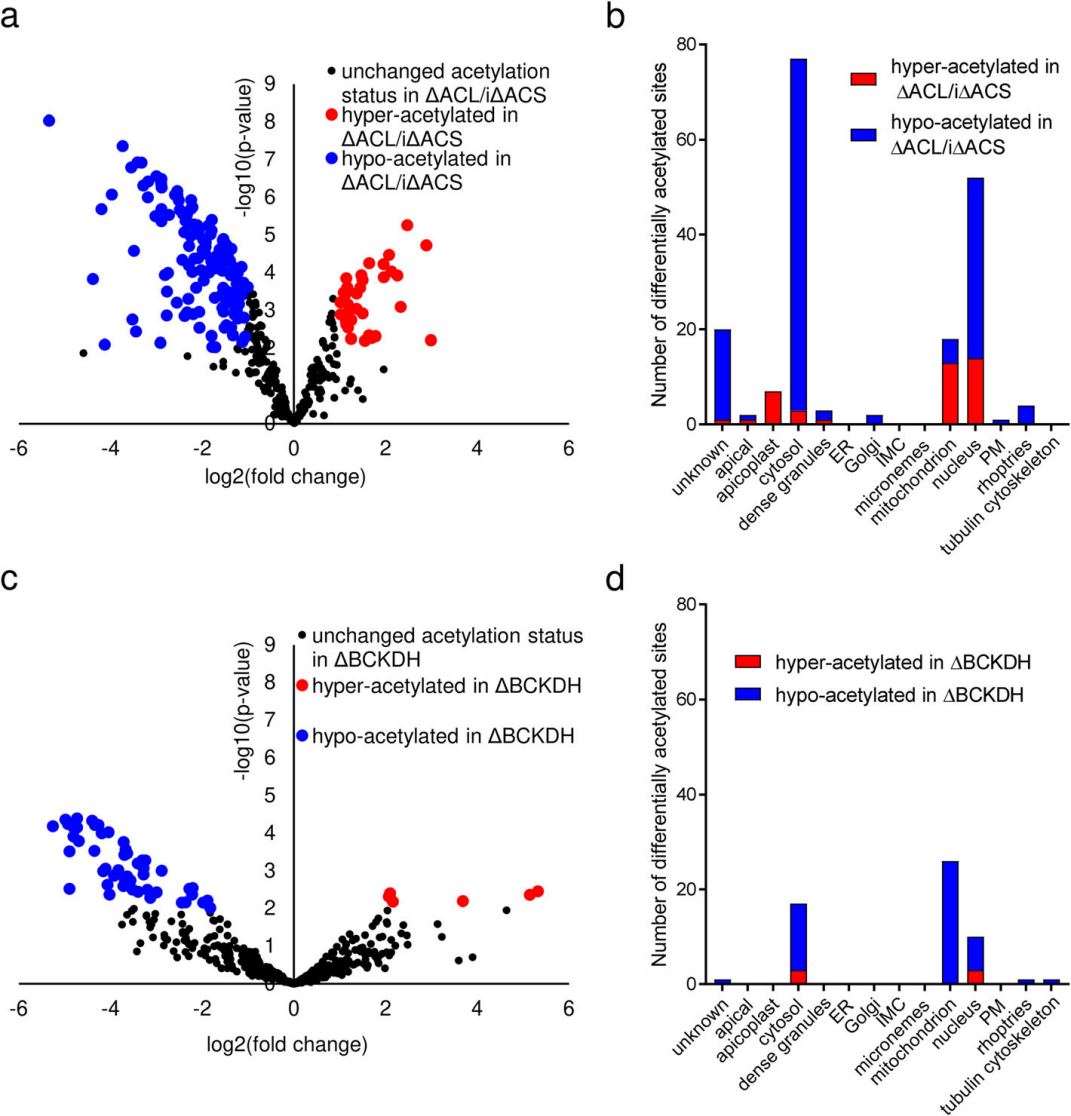
Loss of ACL/ACS or BCKDH results in predominant hypo-acetylation of nucleo-cytosolic and mitochondrial proteins, respectively. **a** Volcano plot highlighting differentially acetylated sites of ΔACL/iΔACS compared to RH parasites. Statistically significant differences between parasite lines were determined as outlined in the ‘Material and methods’ section. Unchanged acetylation sites are displayed in black, while sites which are significantly hyper- or hypo-acetylated in ΔACL/iΔACS (≥ 2-fold, *n* = 3, *limma p* < 0.01) are displayed in red and blue, respectively. **b** Hyper- and hypo-acetylated sites in ΔACL/iΔACS parasites were sorted according to their sub-cellular localisation using the hyperplexed Localisation of Organelle Proteins by Isotopic Tagging (hyperLOPIT) data available under https://proteome.shinyapps.io/toxolopittzex/ [27]. **c** Volcano plot highlighting the differentially acetylated sites in ΔBCKDH compared to RH parasites. Statistically significant differences were determined as outlined in the ‘Material and methods’ section. Unchanged acetylation sites are displayed in black, while sites which are significantly (≥ 2-fold, *n* = 3, *limma p* < 0.01) hyper- or hypo-acetylated in ΔBCKDH are displayed in red and blue, respectively. **d** Hyper- and hypo-acetylated sites in ΔBCKDH parasites were sorted according to their sub-cellular localisation using hyperLOPIT data available under https://proteome.shinyapps.io/toxolopittzex/ [27]. BCKDH, branched-chain α-keto acid dehydrogenase-complex; ACL, ATP citrate lyase; ACS, acetyl-CoA synthetase; PM, plasma membrane; IMC, inner membrane complex; ER, endoplasmic reticulum

To determine the role of cytosolic and mitochondrial acetyl-CoA in protein acetylation, we characterised the acetylome of parasites lacking ACL and ACS or BCKDH, the complex implicated in the production of acetyl-CoA in the mitochondrion [13]. Generation of a parasite line lacking the BCKDH subunit E1 (ΔBCKDH) was described previously [13]. Western blot analysis using α-acetyl-lysine antibodies revealed widespread Nε-lysine-acetylation in *T. gondii* RH, ΔACL/iΔACS and ΔBCKDH parasites (Additional file 5: Figure S4a,b). Quantitative MS-based proteomic analyses comparing RH and ΔACL/iΔACS parasites allowed us to confidently quantify 404 acetylated sites on 269 proteins. Out of these, 182 sites (45%) belonging to 137 proteins were found to be differentially acetylated in ΔACL/iΔACS parasites compared to RH (Additional file 6: Table S2). Most sites (142) were hypo-acetylated while 40 were hyper-acetylated in ΔACL/iΔACS parasites (Fig. 4a). The predominant hypo-acetylation (78% of differentially acetylated sites) is consistent with the expected reduction of acetyl-CoA. The complete dataset is available in a data repository [28].

To determine the sub-cellular localisation of differentially acetylated proteins, we predicted their putative localisation based on the hyperplexed Localisation of Organelle Proteins by Isotopic Tagging (hyperLOPIT) data available under https://proteome.shinyapps.io/toxolopittzex/ and on ToxoDB (https://toxodb.org) [27]. For simplification, we merged some of the sub-cellular compartments (19S proteasome/20S proteasome/40S ribosome/60S ribosome/cytosol into cytosol; nucleolus/nucleus – chromatin/nucleus non-chromatin into nucleus and mitochondrion membranes/mitochondrion soluble into mitochondrion etc.). Differentially acetylated sites were found within proteins of several sub-cellular compartments; however, most hypo-acetylated residues (77%) were of proteins within the cytosol and nucleus, consistent with the localisation of ACS and ACL (Fig. 4b). To account for the different number of proteins within the distinct sub-cellular compartments, we determined the number of differentially acetylated proteins relative to the total number of proteins identified within the compartment, which confirmed that hypo-acetylated proteins were enriched in the cytosol (12% of all cytosolic proteins hypo-acetylated) and nucleus (4.5% of all nuclear proteins hypo-acetylated) (Additional file 5: Figure S4c). Surprisingly, the few sites which were differentially acetylated on proteins in the apicoplast or mitochondrion in ΔACL/iΔACS parasites were predominantly hyper-acetylated (Fig. 4b). The affected hyper-acetylated apicoplast proteins are the PDH, the enoyl-acyl carrier protein reductase (ENR) and a putative chaperone (Additional file 6: Table S2), while the affected mitochondrial proteins are putative chaperones, heat-shock proteins and TCA cycle enzymes (Additional file 6: Table S2). Perhaps, these proteins are hyper-acetylated as part of a stress response to avert the phenotype caused by loss of ACS and ACL. However, the effect of acetylation of these proteins is unknown, and hence, the functional consequences of their hyper-acetylation remain unclear. While ΔACL/iΔACS parasites showed deformation at 24 h of Shld-1 removal, we argue that the observed hypo-acetylation observed in ΔACL/iΔACS parasites at 16 h of Shld-1 removal is a direct result of the loss of acetyl-CoA in the cytosol, rather than a general death phenotype. This is supported by the fact that hypo-acetylation is predominantly observed in the affected cytosol, while other compartments show unaltered acetylation or even hyper-acetylation of proteins (Fig. 4b). Evidence that ΔACL/iΔACS parasites are equally viable and metabolically active as RH parasites at 16 h of Shld-1 removal can also be inferred from the above described metabolomic analyses: e.g. ΔACL/iΔACS parasites show equal rates of FA de novo synthesis (see FA C14:0 levels and labelling in Fig. 3d) as well as equal levels and labelling of most central carbon metabolites (see Additional file 2: Table S1 and Additional file 4: Figure S3a).

As for ΔACL/iΔACS parasites, we probed the acetylome of parasites lacking BCKDH using MS-based quantitative analyses. After stringent filtering, we identified 483 acetylation sites distributed over 300 proteins in RH and ΔBCKDH parasites (Additional file 7: Table S3). Fifty-six lysine residues belonging to 45 different proteins were found to be differentially acetylated in parasites lacking BCKDH compared to RH, with only 6 sites displaying hyper-acetylation and 50 sites being hypo-acetylated (Fig. 4c). Using the hyper-LOPIT data, we identified that most hypo-acetylated residues (52%) belonged to proteins residing within the mitochondrion, consistent with a decreased production of acetyl-CoA within this compartment due to loss of BCKDH catalytic activity (Fig. 4d, Additional file 5: Figure S4d). Taken together, these findings reveal that the lack of ACL/ACS or BCKDH results in predominant hypo-acetylation of numerous proteins within the respective compartment.

### Cytosolic and mitochondrial acetyl-CoA are required for extensive acetylation of glycolytic and TCA cycle enzymes, respectively

**Fig. 5:**
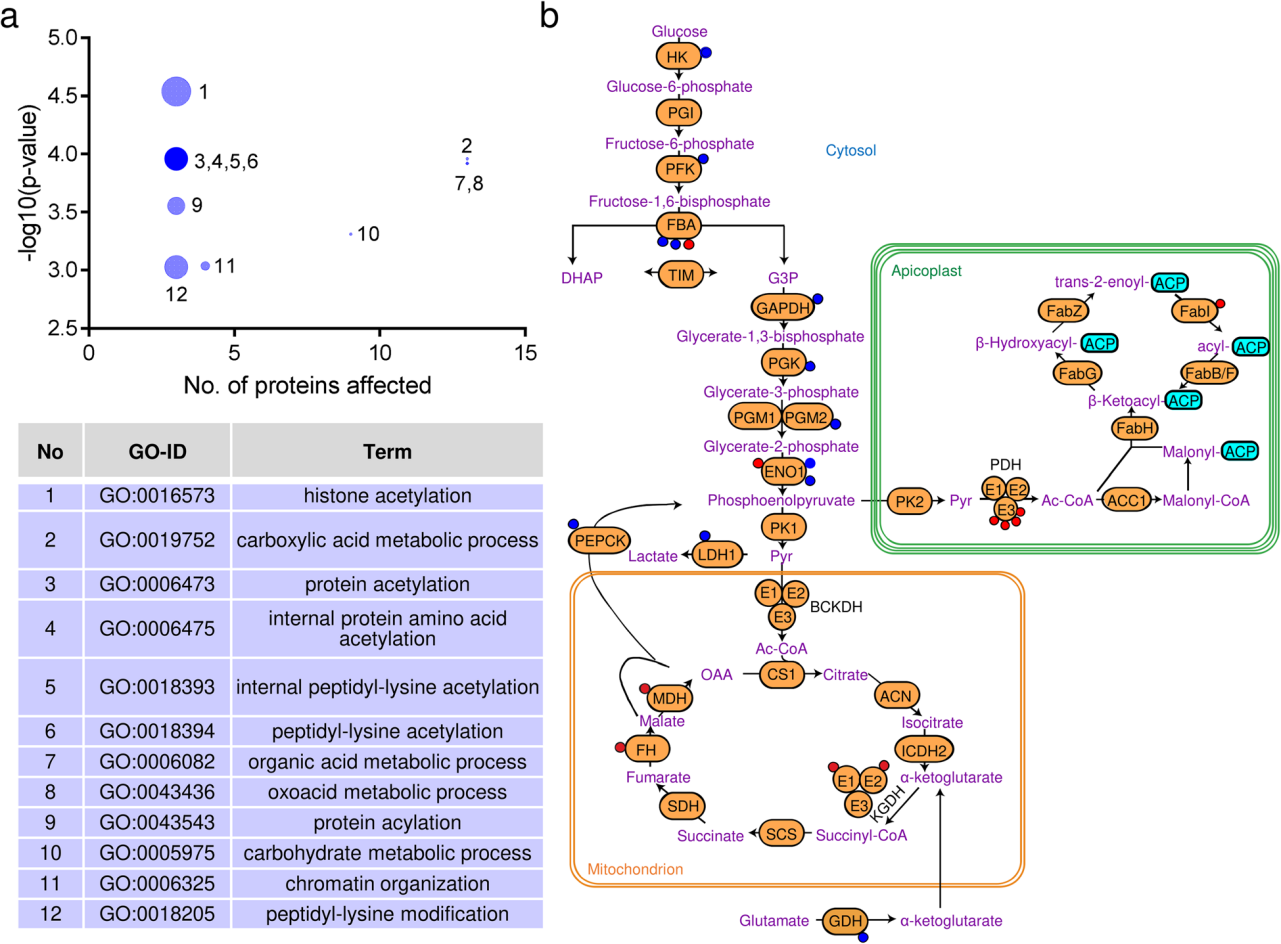
Loss of ACL/ACS causes extensive hypo-acetylation of glycolytic enzymes. **a** Proteins identified as differentially acetylated in ΔACL/iΔACS parasites were analysed using the GO enrichment R-package topGo to identify the enrichment in biological processes against the total *T. gondii* genome/proteome. The bubble sizes are proportional to the fold enrichment, ranging from 33-fold (histone acetylation, 1) to 3-fold (organic acid metabolic process, 7). Statistically significant enrichment was assessed by Fisher’s exact test (*p* value < 0.001). **b** Scheme highlighting the distribution of differentially acetylated sites on metabolic enzymes of ΔACL/iΔACS parasites. Displayed pathways include the glycolysis/gluconeogenesis, the TCA cycle and the apicoplast resident FASII. Coloured circles on the enzymes represent sites which are either hyper- (red) or hypo-acetylated (blue) in ΔACL/iΔACS. ACL, ATP citrate lyase; ACS, acetyl-CoA synthetase; GO, Gene Ontology; TCA, tricarboxylic acid; FASII, fatty acid synthase II; HK, hexokinase; PGI, phosphoglucose isomerase; PFK, phosphofructokinase; FBA, fructose bis-phosphate aldolase; TIM, triosephosphate isomerase; GAPDH, glyceraldehyde 3-phosphate dehydrogenase; PGK, phosphoglycerate kinase; PGM, phosphoglycerate mutase; ENO, enolase; PK, pyruvate kinase; LDH, lactate dehydrogenase; PEPCK, phosphoenolpyruvate carboxykinase-1; BCKDH, branched-chain α-keto acid dehydrogenase-complex; E1-E3, BCKDH subunits; CS, citrate synthase; ACN, aconitase; ICDH, isocitrate dehydrogenase; KGDH, α-ketoglutarate dehydrogenase; E1-E3, KGDH subunits; SCS, succinyl coenzyme A synthetase; GDH, glutamate dehydrogenase; SDH, succinate dehydrogenase; FH, fumarate hydratase; MDH, malate dehydrogenase; PDH, pyruvate dehydrogenase complex; E1-E3, PDH subunits; Ac-CoA, acetyl-CoA; ACP, acyl carrier protein; Fab, FAS II subunits; ACP, acyl carrier protein; ACC, acetyl-CoA carboxylase; Pyr, pyruvate; OAA, oxaloacetate; DHAP, dihydroxyacetone phosphate; G3P, glyceraldehyde-3-phosphate

To better understand the consequences of the differential acetylation in ΔACL/iΔACS and ΔBCKDH parasites, we aimed to identify the affected biological processes by performing Gene Ontology (GO) enrichment using the R-package topGO [29]. Comparing the differentially acetylated proteins to the entire genome/proteome of *T. gondii*, we established that the affected proteins cluster into 12 significantly enriched (*p* < 0.001) biological processes including histone acetylation (33-fold enrichment), chromatin organisation (9-fold enrichment), protein acetylation (19-fold enrichment) and carbohydrate metabolic processes (4-fold enrichment) (Fig. 5a). To avoid bias towards proteins which were identified as acetylated in this study, we performed the GO enrichment analysis of differentially acetylated proteins against the relatively small subset of 269 acetylated proteins as background (Additional file 5: Figure S4e). This analysis revealed the affected proteins to be significantly enriched (*p* < 0.05) in 2 biological processes, namely chromatin organisation and cellular protein metabolic processes. The latter also includes protein modification and is thus consistent with the enrichment of histone and protein acetylation identified in the analysis against the total genome/proteome.

Specifically, the proteins differentially acetylated in ΔACL/iΔACS parasites included several histones (H2Bb, H2Bv, H4, H2AZ) as well as histone-modifying enzymes (histone arginine methyltransferase PRMT1, histone lysine acetyltransferase MYST-A/B, GCN5-A/B, histone acetyltransferase subunit nua4 protein) (Additional file 6: Table S2) [30–33]. Furthermore, we observed considerable hypo-acetylation of the apicomplexan Apetala 2 (AP2) transcription factors AP2XII-4, AP2VIIa-7, AP2IX-7 and AP2IX-5 [34]. Interestingly, two of the differentially acetylated transcription factors, AP2XII-4 and AP2IX-7, have previously been shown to interact with GCN5-B, which is essential for replication [33]. Taken together, these changes in acetylation of histones, histone-modifying enzymes and transcription factors are expected to affect gene expression broadly. Cobbold et al. have previously reported extensive acetylation of AP2 DNA binding proteins in *P. falciparum*, identifying 16 out of 28 AP2 factors to be acetylated in one or more positions [19]. *T. gondii* possesses 67 different AP2 transcription factors [34], 9 of which we identified to be acetylated in one or more positions (Additional file 6: Table S2). AP2XII-4 displayed extensive acetylation in 6 different sites, 3 of which were hypo-acetylated in ΔACL/iΔACS parasites, while 2 were unchanged and one site was hyper-acetylated (Additional file 6: Table S2). In contrast, 2 acetylation sites were identified for its homologue in *P. falciparum*, PF3D7_0516800 [19]. Jeffers and Sullivan have previously identified 5 AP2 domain-containing proteins to be acetylated in *T. gondii* and similarly identified AP2XII-4 to be extensively acetylated in 4 sites [20] compared to 6 sites we report here. A recent ground-breaking study identified the microrchidia (MORC) protein as a key regulator of *T. gondii* development by repressing sexual commitment [35]. Intriguingly, the authors found that MORC functions in a complex with AP2 transcription factors and recruits the histone deacetylase HDAC3 to repress genes which trigger sexual differentiation [35]. Thus, nucleo-cytosolic acetyl-CoA is essential for the regulation of *T. gondii* development by altering histone accessibility and presumably by modifying the activities and interactions of AP2 transcription factors through extensive acetylation.

Besides the hypo-acetylation of nucleo-cytosolic proteins, we also identified hypo-acetylation of four rhoptry proteins (ROP12, ROP17, ROP40 and RON2) and two dense granule proteins (GRA1 and GRA2) (Additional file 6: Table S2), indicating that acetylation of these secretory proteins relies on cytosolic acetyl-CoA, likely imported into the ER by AT-1 [14]. ROPs and GRAs are effector proteins secreted into the host cells during parasite invasion to hijack host cellular functions and to establish and modify the parasitophorous vacuole [36]. Jeffers and Sullivan similarly identified acetylation of ROP 17 and RON2 and proposed additionally acetylation of ROP8 and RON4 but did not detect acetylation of ROP12 [20]. If or how acetylation effects the function of these proteins unique to apicomplexan parasites remains unclear. Nevertheless, hypo-acetylation of these secretory proteins in ΔACL/iΔACS parasites provides the first evidence that their acetylation relies on the cytosolic acetyl-CoA pool.

Next, we analysed the affected metabolic pathways by comparing the differentially acetylated proteins against the entire genome/proteome of *T. gondii* and using the metabolic pathway enrichment tool on ToxoDB (https://toxodb.org/) based on the Kyoto Encyclopedia of Genes and Genomes (KEGG) database as the source. The highest metabolic pathway enrichment was found for proteins functioning in glycolysis/gluconeogenesis (9-fold). Most glycolytic/gluconeogenic enzymes were hypo-acetylated in ΔACL/iΔACS parasites (Fig. 5b). Although enzymes implicated in glycolysis are extensively acetylated in other organisms [37–39], the effects that acetylation has on enzyme activity varies and depends on the organism, enzyme, site and context [37–41]. In the metabolome analysis, we observed no differences in glycolytic flux, i.e. no changes in levels and labelling of most glycolytic intermediates (Additional file 2: Table S1, Additional file 4: Figure S3a), with the exception of a significant reduction in levels of F1,6-BP in ΔACL/iΔACS parasites (Fig. 3b). Strikingly, the enzymes producing and consuming F1,6-BP, phosphofructokinase (PFK) and F1,6-BP aldolase (FBA), respectively, were both differentially acetylated in ΔACL/iΔACS parasites. The differential acetylation may impact on the enzyme’s activity and may cause reduced synthesis or increased consumption of F1,6-BP.

**Fig. 6:**
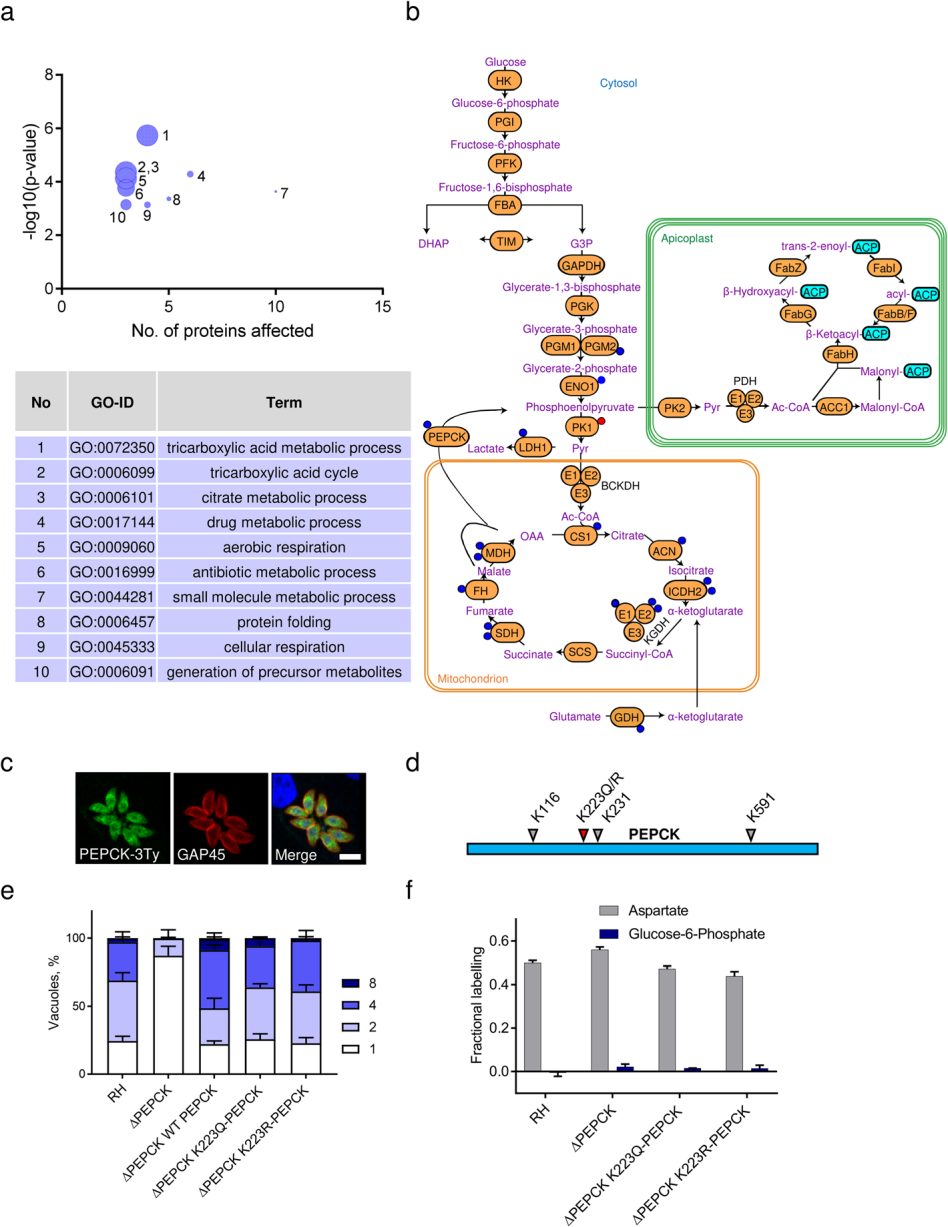
Loss of BCKDH causes extensive hypo-acetylation of TCA cycle enzymes and the gluconeogenic enzyme PEPCK-1. **a** Proteins identified as differentially acetylated in ΔBCKDH parasites were analysed using the GO enrichment R-package topGo to identify enrichment in biological processes against the total *T. gondii* genome/proteome. The bubble sizes are proportional to the fold enrichment of the respective pathway, ranging from 36-fold (GO:0072350—tricarboxylic acid metabolic process) to 4-fold (GO:0044281—small molecule metabolic process). Statistically significant enrichment was assessed by Fisher’s exact test (*p* value < 0.001). **b** Scheme highlighting the distribution of differentially acetylated sites on metabolic enzymes of ΔBCKDH parasites. Displayed pathways include the glycolysis/gluconeogenesis, the TCA cycle and the apicoplast resident FASII. Coloured circles on the enzymes represent sites which are either hyper- (red) or hypo-acetylated (blue) in ΔBCKDH. **c** Endogenous PEPCK-1 presents a nucleo-cytosolic localisation by immunofluorescence assay (IFA) after C-terminal tagging by knock-in of the endogenous *PEPCK-1* locus both in RH parasites. PEPCK-1-3Ty was detected using α-Ty (green) while α-GAP45 (red) was used as a pellicle marker and 4′,6 diamidin-2-phenylindol (DAPI, blue) to stain the nuclei (scale bars in **b** and **c**, 5 μm). **d** Schematic representation of PEPCK and its identified acetylation sites. Lysine at position K223 was changed to glutamine (K223Q acetylation mimetics) or arginine (K223R, de-acetylation mimetics). **e** The ability of different PEPCK-1 acetylation mimetics to grow in a medium lacking glucose was tested in an intracellular growth assay. Error bars represent the standard deviation between 3 independent infections. Per infection, > 100 vacuoles were counted. **f** Constitutive activation of gluconeogenesis was assessed in PEPCK-1 acetylation mimetics by growing cells in a medium containing U-^13^C-glutamine in the presence of unlabelled glucose and measuring ^13^C-labelling in glycolytic intermediates using GC-MS (shown here, glucose-6-phosphate). Uptake and utilisation of U-^13^C-glutamine were confirmed by measuring ^13^C-labelling in the TCA cycle by-product aspartate. Error bars represent the standard deviation between replicates (*n* = 3). Throughout the figure, PEPCK refers to PEPCK-1, the active enzyme in tachyzoites [42]. GO, Gene Ontology; TCA, tricarboxylic acid; FASII, fatty acid synthase II; PEPCK, phosphoenolpyruvate carboxykinase-1; BCKDH, branched-chain α-keto acid dehydrogenase-complex; GC-MS, gas chromatography-mass spectrometry; TCA, tricarboxylic acid; other abbreviations, see Fig. 5

As described above for ΔACL/iΔACS parasites, we performed the GO enrichment analysis of biological processes and metabolic pathways of proteins differentially acetylated in ΔBCKDH parasites. Differentially acetylated proteins in ΔBCKDH parasites were compared to the total genome/proteome using the R-package topGO [29], which identified affected proteins to be significantly enriched (*p* < 0.001) in 10 biological processes, most of which are related to the TCA cycle and respiration, including the top hits with over 30-fold enrichment (tricarboxylic acid metabolic processes, tricarboxylic acid cycle and citrate metabolic processes) (Fig. 6a). In order to avoid bias towards acetylated proteins, differentially acetylated proteins were probed against the background of the small subset of acetylated proteins. Using this approach, 6 biological processes were identified as significantly enriched (*p* < 0.05) all of which related to the TCA cycle and respiration and 5 of which were also identified in the enrichment analysis against the entire genome/proteome (Additional file 5: Figure S4f). As expected, analysis of proteins which were differentially acetylated in ΔBCKDH parasites revealed major enrichment in the TCA cycle when using the metabolic pathway enrichment tool on ToxoDB (https://toxodb.org/) based on the KEGG database as the source, returned the TCA cycle as top-hit (24-fold enrichment). Seven out of eight TCA cycle enzymes were hypo-acetylated in ΔBCKDH parasites compared to RH cells (Fig. 6b). As for glycolytic enzymes, the role of TCA cycle enzyme acetylation varies and has not been defined for *T. gondii* enzymes [41, 43].

One of the most significantly hypo-acetylated proteins in ΔBCKDH cells was lactate dehydrogenase (LDH1) (Additional file 7: Table S3). Acetylation in lysine K5 has been reported to negatively regulate LDH activity in mammalian cells [44]. Although we observed hypo-acetylation of a different lysine (K218) in ΔBCKDH parasites, it may similarly increase LDH activity, as lactate production is markedly increased in ΔBCKDH parasites [13].

Furthermore, we revealed a previously unpublished acetylation site in the gluconeogenic enzyme phosphoenolpyruvate carboxykinase (PEPCK-1) (K116) in addition to the previously identified acetylation sites K223, K231 and K591 of *T. gondii* PEPCK-1 [20] (Additional file 7: Table S3). Interestingly, lysine residue K223 was found to be significantly hypo-acetylated in ΔBCKDH parasites (Additional file 7: Table S3, Fig. 6b). We previously reported constitutive activation of the gluconeogenesis pathway in ΔBCKDH parasites [13], leading us to hypothesise that the activation of gluconeogenesis in ΔBCKDH parasites may be attributed to the changed acetylation status of PEPCK-1 (Additional file 8: Figure S5a).

### The PEPCK-1 acetylation status is not solely responsible for regulating gluconeogenesis in *T. gondii*

In *T. gondii*, gluconeogenesis is tightly regulated and inactive under glucose replete conditions [45, 46] but essential in glucose-limiting conditions [42, 47]. *T. gondii* possess two PEPCKs, with PEPCK-1 (TGME49_289650) being the active enzyme in tachyzoites [42]. We identified two in-frame translation starts for PEPCK-1 (Additional file 8: Figure S5b). While the long isoform of PEPCK-1 localises to the mitochondrion (Additional file 8: Figure S5c), a second ATG, 285 bases downstream of the first predicted translational start, leads to a shorter isoform with cytosolic localisation (Additional file 8: Figure S5c). Crucially, a 3-Ty-epitope tag at the C-terminal end of the endogenous gene locus (Additional file 8: Figure S5d,e) revealed cytosolic localisation of the endogenous PEPCK-1 (Fig. 6c), contrasting previous reports, which have proposed a mitochondrial localisation [42]. To test whether the constitutive activation of gluconeogenesis in ΔBCKDH parasites [13] is due to hypo-acetylation of PEPCK-1 K223, we complemented ΔPEPCK-1 parasites with wild-type PEPCK-1 or versions of PEPCK-1 mimicking acetylation (glutamine) or de-acetylation (arginine) of lysine at position 223 (K223Q, K223R) (Fig. 6d). The *pepck*-1 locus was deleted by double homologous recombination (Additional file 8: Figure S5f). Its deletion and insertion of the chloramphenicol acetyltransferase (CAT) resistance cassette were confirmed by genomic PCR (Additional file 8: Figure S5g). Expression of the acetylation mimetic PEPCK-1 constructs was confirmed by IFA (Additional file 8: Figure S5h).

To investigate whether gluconeogenesis was constitutively active or inactive depending on the PEPCK-1 acetylation status, ΔPEPCK-1 strains complemented with PEPCK-1 acetylation mimetics (K223Q, K223R) were assessed in a growth assay in glucose-depleted medium (Fig. 6e). The acetylation/de-acetylation mimetics of PEPCK-1 (K223Q/K223R) grew equally well and comparable to parasites complemented with WT-PEPCK-1 in glucose-depleted medium (Fig. 6e). Only parasites lacking PEPCK-1 showed a growth defect in the glucose-depleted medium. These findings indicate that acetylation of PEPCK-1 (K223Q) alone is not sufficient to de-activate gluconeogenesis. To assess the constitutive activation of gluconeogenesis, PEPCK-1 acetylation mimetic parasites were incubated in a medium containing unlabelled glucose and U-^13^C-glutamine followed by profiling of polar metabolites by GC-MS (Fig. 6f). Labelled carbons from U-^13^C-glutamine were not incorporated into glycolytic/gluconeogenic intermediates, such as glucose-6-phospate, in the presence of unlabelled glucose (Fig. 6f), as was previously observed in ΔBCKDH parasites [13], highlighting that activation of gluconeogenesis is not triggered by de-acetylation of PEPCK-1 K223 (K223R) alone. Hence, the impact of acetylation on the function of enzymes in *T. gondii* central carbon metabolism remains unclear. Lastly, we assessed whether gluconeogenesis was an important adaptation in parasites lacking BCKDH. As described above for RH, *pepck*-1 was deleted in parasites lacking BCKDH (Additional file 8: Figure S5i). Strikingly, ΔBCKDH/ΔPEPCK-1 parasites showed no aggravation of their growth phenotype compared to parasites lacking either BCKDH or PEPCK-1 as assessed by plaque assay and intracellular growth assay in the presence and absence of glucose (Additional file 8: Figure S5j-l). These results highlight that the activation of gluconeogenesis in ΔBCKDH parasites is a ‘side effect’ rather than a crucial adaptation mechanism.

### Lack of ACL/ACS or BCKDH alters the transcriptome and proteome of *T. gondii*

**Fig. 7:**
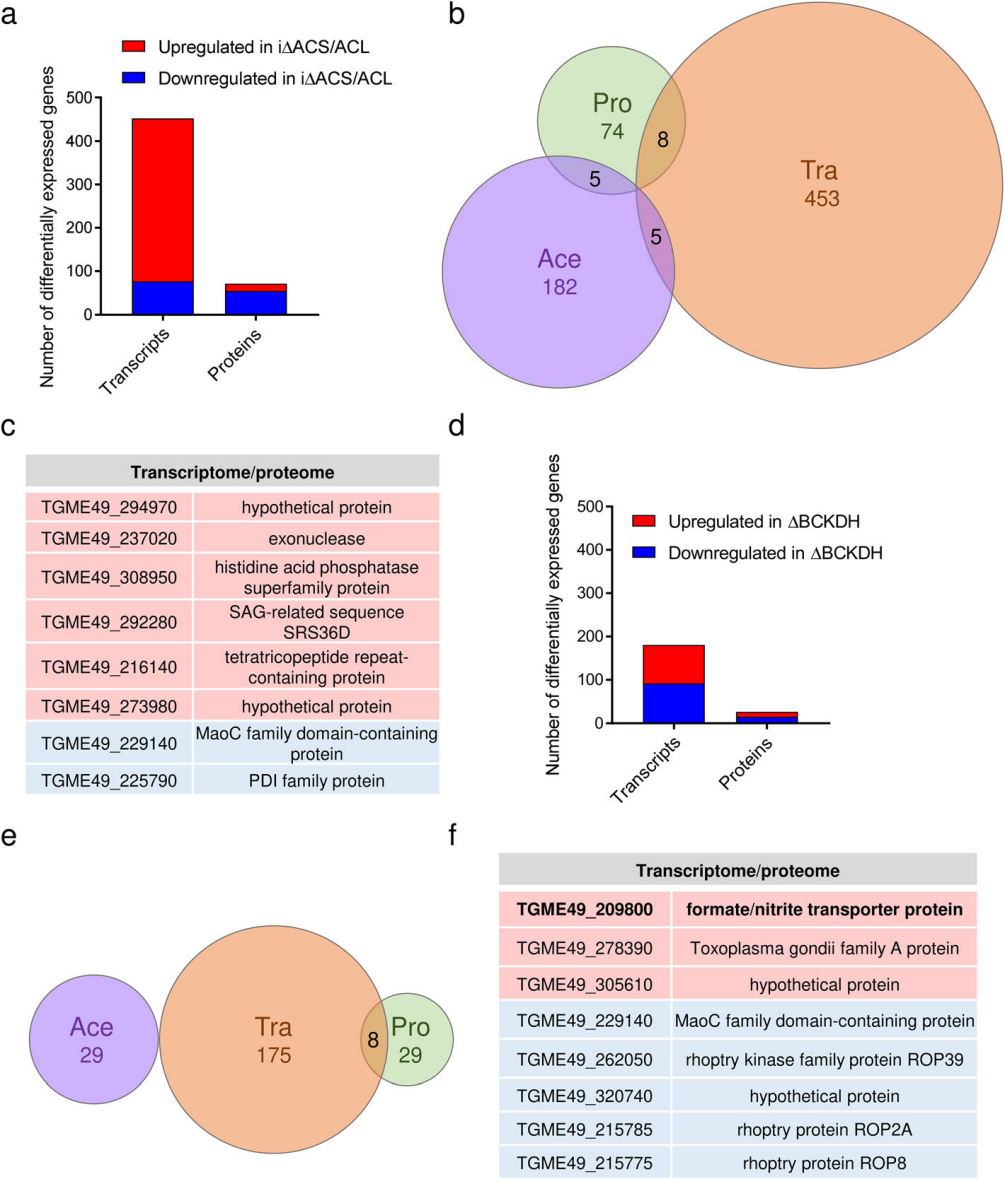
Loss of ACL/ACS or BCKDH alters the *T. gondii* gene expression. **a** Graph showing the number of RNA transcripts and proteins which are ≥ 2-fold up- (red) or downregulated (blue) in ΔACL/iΔACS parasites compared to RH (*n* = 3; *limma p* < 0.01). Statistical significance was determined as outlined in the ‘Material and methods’ section. **b** Venn diagram highlighting the overlap between differentially expressed RNA transcripts (Tra), proteins (Pro) and differentially acetylated proteins (Ace) which present a ≥ 2-fold change in ΔACL/iΔACS parasites compared to RH parasites (all *p* < 0.01). **c** Table highlighting genes which were found to be significantly up- (red background) and downregulated (blue background) at the transcriptome and proteome level in ΔACL/iΔACS parasites. **d** Graph highlighting the number of RNA transcripts and proteins which are ≥ 2-fold up- (red) or downregulated (blue) in ΔBCKDH parasites compared to RH (*n* = 3; *limma p* < 0.01). Statistical significance was determined as outlined in the ‘Material and methods’ section. **e** Venn diagram highlighting the overlap between differentially regulated acetylation sites (Ace), RNA transcripts (Tra) and proteins (Pro), which present a > 2-fold change (*p* < 0.01) in ΔBCKDH parasites compared to RH parasites. **f** Table highlighting genes which were found to be significantly up- (red background) and downregulated (blue background) at the transcriptome and proteome level in ΔBCKDH parasites. BCKDH, branched-chain α-keto acid dehydrogenase-complex; ACL, ATP citrate lyase; ACS, acetyl-CoA synthetase; RNA, ribonucleic acid

For a global assessment of the impact caused by the depletion of cytosolic acetyl-CoA on gene expression in ΔACL/iΔACS parasites, we performed a combination of expression profiling by ribonucleic acid sequencing (RNA-seq) and quantitative proteomics analyses on freshly egressed extracellular tachyzoites. The complete datasets are available in data repositories [28, 48]. We focused our analysis on transcripts and proteins that were changed ≥ 2-fold in their level (*p* value < 0.01) in ΔACL/iΔACS parasites compared to RH. RNA-seq analysis allowed the identification of 453 differentially regulated transcripts out of 7250; 377 were found to be upregulated while 76 were downregulated (Fig. 7a, Additional file 9: Table S4). The predominant upregulation of transcripts in parasites depleted in nucleo-cytosolic acetyl-CoA was surprising, considering that histone de-acetylation is associated with a general downregulation of transcription [7]. As anticipated, the HXGPRT resistance cassette transcript belonged to the top upregulated hits while ACL was amongst the top hits of downregulated transcripts, validating the obtained results. Transcripts or proteins influenced directly by the experimental procedure were excluded from further analysis. Of the differentially acetylated transcripts, 284 encoded hypothetical proteins, hindering interpretation of the results. The remaining transcripts encoded for proteins of diverse functions and included several *T. gondii*-specific proteins such as surface antigen (SAG)-related proteins (16 transcripts) and *Toxoplasma gondii* family (A,B,C) proteins, a group of uncharacterised *T. gondii*-specific proteins (14 transcripts). GO enrichment against the entire genome/proteome of *T. gondii* using the biological process enrichment tool on ToxoDB (https://toxodb.org/) revealed no significant enrichment of the affected transcripts in biological processes.

MS-based proteomics allowed identification and relative quantification of 2773 *T. gondii* proteins, out of which 74 were found to be differentially expressed in ΔACL/iΔACS parasites compared to RH (Fig. 7a, Additional file 10: Table S5). In contrast to the transcriptome, most proteins [49] were downregulated in ΔACL/iΔACS parasites (Fig. 7a, Additional file 10: Table S5). Amongst the downregulated proteins were ACS and ACL, while the HXGPRT selection cassette was amongst the top hits of upregulated proteins, validating the obtained data (Additional file 10: Table S5). The altered proteins have highly variable functions and include 26 hypothetical proteins, hindering the interpretation of the results.

Myosin J (MyoJ), a myosin motor mediating constriction of the basal pole during parasite division [50], was found to be downregulated over 2-fold (*p* value < 0.01) at the protein level in ΔACL/iΔACS strain compared to RH (Additional file 10: Table S5). Loss of MyoJ has been shown to be associated with a loss of fitness in *T. gondii*, resulting in asynchronous division and the loss of connection between daughter cells [50]. Hence, its downregulation may contribute to the severe morphological defects of ΔACL/iΔACS parasites described above (Fig. 2).

A relatively small subset of 8 genes was affected simultaneously at the transcriptome and proteome level, with 2 genes being downregulated at the RNA transcript and protein level, while 6 were upregulated at both levels (Fig. 7b, c). This relatively small overlap between the datasets highlights the diverse and complex consequences of cytosolic acetyl-CoA depletion.

Similarly, expression profiling by RNA-seq and quantitative proteomics was performed on freshly egressed ΔBCKDH tachyzoites to obtain a more holistic assessment of the consequences of mitochondrial acetyl-CoA depletion. Transcriptomic analysis revealed that a relatively small subset of transcripts (175 out of 6926) was differentially expressed (≥ 2-fold change, *p* value < 0.01) in ΔBCKDH compared to RH parasites (Fig. 7d, Additional file 11: Table S6) with 90 transcripts being down- and 85 being upregulated. Upregulation of the HXGPRT resistance cassette transcripts and downregulation of BCKDH subunit E1 transcripts provided confidence in the results. Of the differentially expressed transcripts, 100 encode hypothetical proteins hindering the interpretation of the results. GO enrichment analysis using the biological process enrichment tool on ToxoDB (https://toxodb.org/) against the total *T. gondii* genome/proteome indicated a significant enrichment of differentially expressed transcripts in cell differentiation (*p* < 0.05) although only 2 transcripts were affected (Additional file 11: Table S6). Many transcripts encode *T. gondii*-specific proteins such as SAG-related proteins (12 transcripts) and *Toxoplasma gondii* family proteins (6 transcripts) (Additional file 11: Table S6).

MS-based proteomics allowed identification and relative quantification of 2242 *T. gondii* proteins. As for the transcriptome, a relatively small subset of proteins [30] was found to be differentially expressed (≥ 2-fold change, *p* value < 0.01) in ΔBCKDH parasites, out of which 16 were downregulated in the mutant parasites while 13 were upregulated (Fig. 7d, Additional file 12: Table S7). Downregulation of BCKDH-E1 and upregulation of HXGPRT validated the obtained data (Additional file 12: Table S7). The differentially expressed proteins included 8 hypothetical proteins and proteins of diverse functions. GO term enrichment analysis of biological processes (limited to GO slim terms) against the entire genome/proteome of *T. gondii* using the tool on ToxoDB indicated significant enrichment (*p* < 0.05) in carbohydrate metabolic processes and cellular protein modification processes, with 3 and 4 proteins affected, respectively (Additional file 12: Table S7). Comparison of the different datasets revealed no overlap of the altered acetylome with either the transcriptome or proteome. However, 8 genes were differentially expressed simultaneously at the transcript and protein level (Fig. 7e, f), 3 of which were significantly upregulated at the transcript and protein level, while 5 were downregulated at both levels, including 3 rhoptry proteins, which are secreted during parasite invasion [36] (Fig. 7f).

**Fig. 8:**
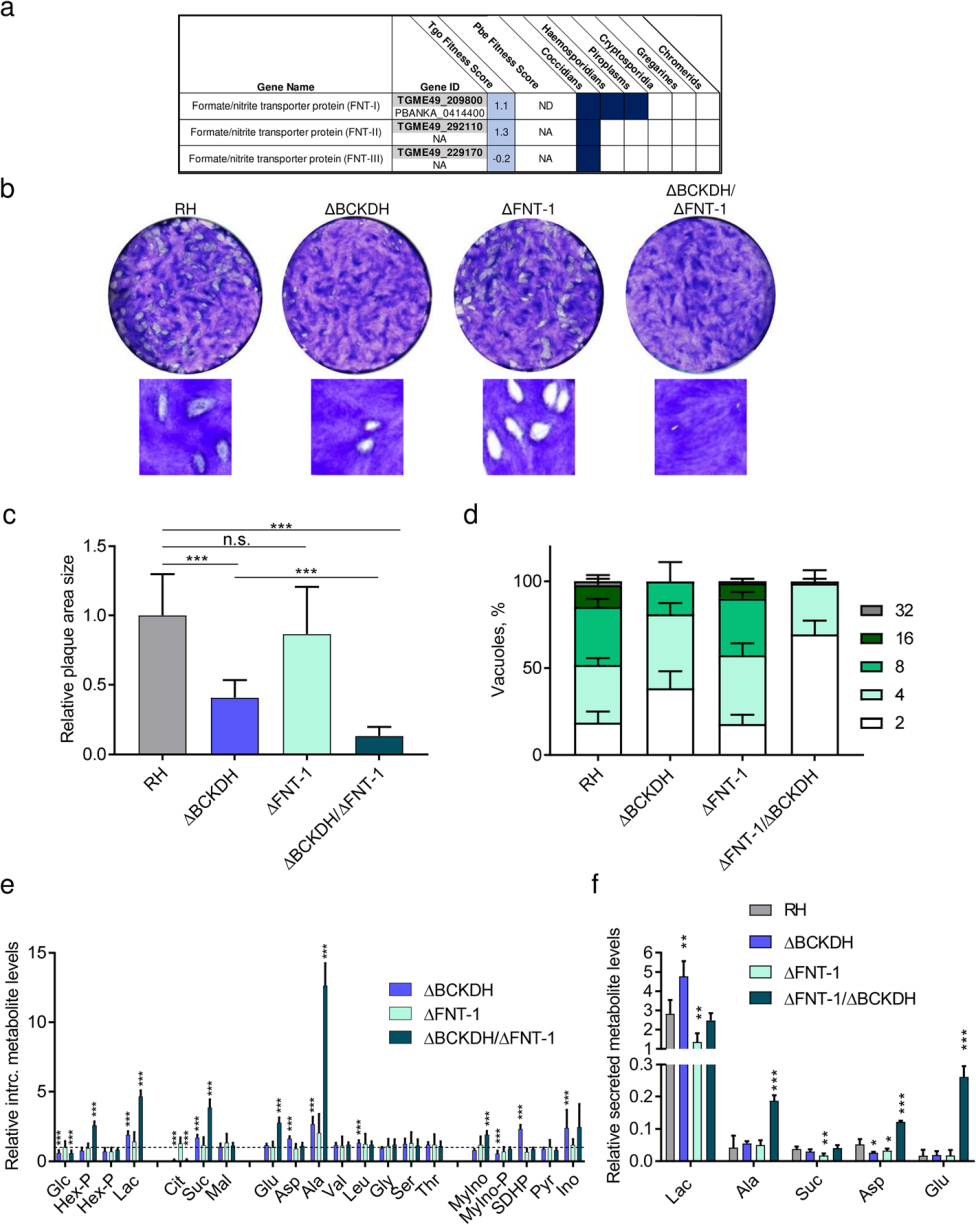
FNT-1 is dispensable in wild-type *T. gondii* but becomes highly fitness-conferring in ΔBCKDH parasites. **a** Table highlighting the conservation of formate/nitrite transporters (FNTs) across apicomplexans and their fitness score from a recent screen fitness screen of metabolic genes. **b** Plaque assay, testing the fitness during the lytic cycle of RH parasites and mutant parasites lacking BCKDH, FNT-1 or both. **c** Quantification of plaque area size comparing different strains: RH, ΔBCKDH, ΔFNT-1 and ΔBCKDH/ΔFNT-1 double-KO parasites. Error bars represent the standard deviation between 3 independent infections. Per infection, the areas of > 20 plaques were quantified, and statistically significant differences were determined by a *t*-test comparing the mutants as indicated (n.s., non-significant; ***p < 0.001). **d** Intracellular growth assay of RH and mutant parasites over 36 h. Error bars represent the standard deviation between 3 independent infections. Per infection, > 100 vacuoles were counted. **e**, **f** Metabolomic analysis of *T. gondii* RH and mutant parasites (ΔBCKDH, ΔFNT-1 and ΔBCKDH/ΔFNT-1). Levels of intracellular metabolites relative to levels in RH (dashed line) (**e**) and of metabolites secreted into the medium by purified parasites normalised to valine, an essential amino acid present in the culture medium at 0.8 mM (**f**). Error bars in **e** and **f** represent the standard deviation between replicates (*n* = 4). Statistically significant differences between each mutant and RH were assessed using a *t*-test and are indicated (*p < 0.05; **p < 0.005; ***p < 0.001). BCKDH, branched-chain α-keto acid dehydrogenase-complex; Glc, glucose; Hex-P, hexose-phosphate; Lac, lactate; Cit, citrate; Suc, succinate; Mal, malate; Glu, glutamate; Asp, aspartate; Ala, alanine; Val, valine; Leu, leucine; Gly, glycine; Ser, serine; Thr, threonine; MyIno, myo-inositol; MyIno-P, myo-inositol-phosphate; SDHP, sedoheptulose-7-phosphate; Pyr, pyrimidine; Ino, inosine

One gene, for which expression was upregulated over 2-fold at the transcript and protein levels in ΔBCKDH parasites, belonged to the formate/nitrite transporter (FNT) family (TGME49_209800, FNT-1) (Fig. 7f). In coccidians, three members of this protein family catalyse the transport of monocarboxylate metabolites such as lactate (Fig. 8a) [51]. In contrast, haemosporidians and piroplasms express a single FNT, which has been shown to be essential in *Plasmodium* and is the target of several antimalarials [52–54]. *T. gondii* parasites lacking BCKDH have previously been shown to have increased levels of intracellular pyruvate and lactate due to the disruption of the link between glycolysis and the TCA cycle [13]. Under these circumstances, lactate secretion may be increasingly important to maintain metabolic homeostasis.

### FNT-1 is dispensable in RH but becomes fitness-conferring in ΔBCKDH parasites

To assess whether the upregulation of FNT-1 is important for the metabolic adaptation in ΔBCKDH cells, we deleted *fnt-1* in RH and ΔBCKDH parasites. Using CRISPR-Cas9, we replaced the *fnt-1* locus with a dihydrofolate reductase-thymidylate synthase (*dhfr-ts*) resistance cassette (Additional file 13: Figure S6a). Integration of the resistance cassette and the absence of the original gene locus were confirmed by PCR in the single- and double-knock-out (KO) (Additional file 13: Figure S6b). Assessment of the fitness of the different strains by plaque assay confirmed that ΔBCKDH parasites present a modest but significant reduction in plaque size (Fig. 8b, c), as previously reported [13]. In contrast, ΔFNT-1 parasites formed plaques of normal sizes comparable to RH cells, while ΔBCKDH/ΔFNT-1 parasites formed very small plaques, which were considerably smaller than those of ΔBCKDH cells (Fig. 8b,c). An intracellular growth assay confirmed the normal development of ΔFNT-1 cells and the aggravated phenotype of ΔBCKDH/ΔFNT-1 compared to ΔBCKDH cells (Fig. 8d). An overview of the growth and fitness defects of the various strains analysed in this study is provided (Additional file 13: Figure S6c).

To characterise the defect in the metabolism of ΔBCKDH/ΔFNT-1 parasites, intracellular metabolites involved in central carbon metabolism were profiled by GC-MS (Fig. 8e). Interestingly, none of the detected metabolites was significantly altered in ΔFNT-1 parasites compared to RH. Instead, ΔBCKDH parasites displayed significant changes such as increased lactate and markedly reduced citrate consistent with our previous analysis [13]. ΔBCKDH/ΔFNT-1 presented an aggravation of the metabolic phenotype observed in ΔBCKDH parasites (Fig. 8e). The observed 5-fold increase in intracellular lactate in ΔBCKDH/ΔFNT-1 (compared to 2-fold in ΔBCKDH) likely impacts on the parasite fitness [51, 53, 55]. To specifically test whether ΔFNT-1 and ΔBCKDH/ΔFNT-1 parasites displayed defective lactate secretion, we measured the levels of metabolites secreted into the medium by the different *T. gondii* strains (Fig. 8f). Secreted metabolites detected in the medium included lactate, alanine, succinate, aspartate and glutamate, consistent with previous studies [46]. ΔBCKDH cells displayed the highest secretion of lactate, while ΔFNT-1 secreted the lowest levels of lactate (Fig. 8f). ΔBCKDH/ΔFNT-1 exported lactate at similar levels as RH but secreted markedly higher levels of alanine, aspartate and glutamate (Fig. 8f). Our findings highlight that lactate is exported in cells devoid of ΔFNT-1, consistent with the presence of other lactate transporters FNT-2 and FNT-3 [51]. However, secretion of lactate is significantly decreased in ΔFNT-1 compared to RH cells and in ΔBCKDH/ΔFNT-1 compared to ΔBCKDH parasites, confirming a prominent role of FNT-1 in efficient lactate secretion. Unable to fuel pyruvate after conversion to acetyl-CoA into the TCA cycle and secreting lactate inefficiently, these cells accumulate toxic intracellular levels of lactate and alanine. Our data highlight the metabolic flexibility of *T. gondii* to adapt to obstructions at the transcriptional, translational and post-translational levels.

## Discussion

Enzymes generating acetyl-CoA, as well as enzymes involved in modulating histone acetylation (histone acetylases and deacetylases), have been proposed as drug targets in apicomplexan parasites [17, 32, 56] and play a crucial role in *T. gondii* development [35]. However, we know little about how acetylation regulates gene expression and enzyme activities and how acetyl-CoA contributes to the metabolism in these pathogens. We reveal here that cytosolic acetyl-CoA affects the levels of various transcripts and proteins. Additionally, we demonstrate that it is required for the elongation of FAs. *T. gondii* synthesises monounsaturated long and very long-chain FAs (FA C20:1, C26:1, C28:1), which are of low abundance or absent in the host cell, making the FA elongation pathway in *T. gondii* essential [25]. While a previous study reported that depletion of ACS impacts on the FA elongation pathway in *T. gondii*, the crucial monounsaturated very long-chain FAs (FA C26:1, C28:1) were not detected in the study by Dubois and colleagues, and the phenotype was very modest given the compensatory effect of ACL [57]. FAs generated by FASII as well as their derivatives generated through the elongation pathway have a fundamental role for the completion of *T. gondii* cytokinesis and pellicle formation between the emerging daughter cells [49]. Hence, inhibition of the FA elongation pathway may partially contribute to the described amorphic phenotype of cells devoid of ACS and ACL.

In contrast to the essential pool of nucleo-cytosolic acetyl-CoA, loss of mitochondrial acetyl-CoA can be tolerated by *T. gondii* at a modest fitness cost [13]. We reveal here that lack of BCKDH causes relatively few specific changes in the acetylome, transcriptome and proteome, some of which are crucial to enable these parasites to tolerate obstruction of the link between glycolysis and the TCA cycle. MacRae et al. have proposed that the portion of pyruvate entering the TCA cycle in *T. gondii* is about 20% under regular culture conditions [46], while the majority is secreted as lactate, through FNT-1 or FNT-2, the two lactate transporters expressed in tachyzoites [51]. In contrast, *Plasmodium* parasites, which rely on glycolysis during their intraerythrocytic development, express a single FNT, depletion/inhibition of which leads to parasite death, due to the toxic accumulation of lactate [52, 53]. In *T. gondii*, a genome-wide fitness screen using CRISPR-Cas 9 reported low positive fitness indices for FNT-1 (1.10) and FNT-2 (1.31), suggesting that both genes are individually dispensable [11]. Indeed, we were able to deplete FNT-1 in tachyzoites without causing any fitness defect. Importantly, these parasites continued to secrete lactate, highlighting that FNT-2 secretes lactate sufficiently under normal culture conditions, compensating for the loss of FNT-1. However, depletion of FNT-1 in cells devoid of BCKDH resulted in a severe additional fitness defect, and double-KO parasites accumulated very high levels of intracellular lactate. We propose that the identified hypo-acetylation of LDH1, as well as the overexpression of FNT-1 in cells lacking BCKDH, is part of a coping mechanism which enables increased reliance on glycolysis in the absence of a functional TCA cycle. These findings provide insights into how parasites adapt their metabolism in response to genetic or pharmacological obstructions.

## Conclusions

This study combines molecular tools and multi-omics to provide a uniquely integrative and global picture of the diverse roles of acetyl-CoA in *Toxoplasma gondii* physiology. We demonstrate that loss of nucleo-cytosolic acetyl-CoA results in hypo-acetylation of histones and non-histone proteins causing broad changes in gene expression. Further, we show that the absence of cytosolic acetyl-CoA results in a halt in FA elongation, disabling the synthesis of parasite-specific long-chain monounsaturated FAs which cannot be salvaged from the host.

In contrast to the cytosolic acetyl-CoA pool, loss of mitochondrial acetyl-CoA can be tolerated due to an altered central carbon metabolism [13]. How protozoan parasites remodel their metabolism to adapt to varying environments or to obstructions through genetic alterations or drug treatments is poorly understood. We demonstrate here that loss of mitochondrial acetyl-CoA results in the hypo-acetylation of mitochondrial and other proteins as well as in altered gene expression. We provide evidence that these changes at the transcriptional, translational and post-translational levels serve to adapt the metabolism and cope with the lack of mitochondrial acetyl-CoA. These findings provide unprecedented insights into the plasticity and regulation of the metabolism of *T. gondii*.

## Material and methods

### Genetic and cell biology approaches

#### *T. gondii* culture

All *T. gondii* strains are derived from RH in which KU80 has been deleted [58]. Parasites were grown in confluent HFFs and maintained in Dulbecco’s modified Eagle medium (DMEM, Life Technology, Invitrogen) supplemented with 5% foetal calf serum, 2 mM l-glutamine, 25 μg/ml gentamicin and where indicated with 0.5 μM Shld-1 [23] in a humidified incubator at 37 °C and 5% CO_2_. Specific media for stable isotope labelling experiments are described below.

#### Cloning of DNA constructs

Amplifications of DNA fragments for cloning were performed with either the LA Taq (TaKaRa) or the Q5 (New England Biolabs) polymerases, and the primers used for each reaction are listed in Additional file 14: Table S8. Correct integration of the different constructs into the genome of the various strains was determined by genomic PCR using the GoTaq Green Master Mix (Promega) and the primers listed in Additional file 14: Table S8.

#### Preparation of *T. gondii* genomic DNA

Genomic DNA was extracted from extracellular tachyzoites using the Wizard SV genomic DNA purification system (Promega).

#### Inducible knock-down of ACS

To direct the insertion of this PCR product, a specific guide RNA (gRNA) vector targeting the ATG start codon of *acs* (TGME49_266640) was generated using the Q5 site-directed mutagenesis kit (New England Biolabs) and the vector pSAG1::CAS9-GFP-U6::sgUPRT as a template [59]. The UPRT-targeting gRNA was replaced by an *acs*-specific gRNA using the primer pair 1-2 listed in Additional file 14: Table S8. A PCR fragment was amplified of the DD, fused to a myc-tag (DDmyc) with 5′ and 3′ homology sequences to the start ATG of ACS using primers P7/P9 as shown in Additional file 1: Figure S1a,c. (KOD polymerase, Novagen) and pTub8DDmycROM4 as template [60]. Ten micrograms of the gRNA plasmid, together with the precipitated KOD PCR product, was transfected, and parasites were grown in the presence of Shld-1. Parasites expressing the Cas9-GFP were sorted by fluorescence-activated cell sorting (FACS) and cloned into 96-well plates using a cell sorter (MoFlo Astrios, Beckman Coulter). Clones were analysed by IFA to confirm the expression of the DDmycACS fusion protein and grown in the presence or absence of Shld-1 to evaluate the regulation of the protein.

#### Knock-in construct for epitope tagging at the endogenous locus of *pepck-1*

A genomic DNA fragment of the C-terminal part of *pepck-1* (TGME49_289650) was amplified by PCR using the primer pair 3-4 listed in Additional file 14: Table S8, digested with the restriction enzymes KpnI/SbfI and cloned into the pTUB8MIC13-3Ty-HX [61], using the KpnI and NsiI sites. Prior to transfection, the plasmid was linearised in the middle of the cloned genomic DNA fragment using the NcoI site. For knock-in insertion of these vectors into the RH and ΔBCKDH strains, the *hxgprt* cassette was substituted with a *dhfr-ts* cassette using the two SacII sites.

#### Generation of PEPCK-1 second copy-expressing plasmids and acetylation mimetics

pTub8-PEPCK-1-L-3Ty and pTub8-PEPCK-1-S-3Ty plasmids expressing a second copy of both long and short isoforms of PEPCK-1, respectively, were generated by amplifying the cDNA of *pepck-1* using the primers 9-4 (long) or primers 4-10 listed in Additional file 14: Table S8, digested with the restriction enzymes EcoRI and SbfI and cloned into the KpnI and NsiI sites of pTub8-APHN21-3Ty-HXGPRT [62]. To complement ΔPEPCK-1 with either a wild-type copy of PEPCK-1, acetylation mimetics or de-acetylation mimetics of PEPCK-1, 5’UPRT-DHFR-pTub8-PEPCK-1-4myc-3’UPRT was first generated. 5’UPRT-DHFR-pTub8-PEPCK-1-4myc-3’UPRT was generated by amplifying the cDNA of *pepck-1* using the primers 4-10 listed in Additional file 14: Table S8, digested with the restriction enzymes EcoRI and SbfI and cloned into the of the pUPRT-pTub8-4myc-3’UPRT plasmid. A *dhfr-ts* cassette was amplified by PCR using primers 11-12, digested by ApaI, sub-cloned into pUPRT-pTub8-PEPCK-1-4myc-3’UPRT and predigested by ApaI. Lysines K223, K231 and K591 were mutated to either arginine (de-acetylation mimetic) or glutamine (acetylation mimetic) using the Q5 site-directed mutagenesis kit (New England Biolabs), primer pairs listed in Additional file 14: Table S8 and 5’UPRT-DHFR-pTub8-PEPCK-1-4myc-3’UPRT as a template. 5’UPRT-DHFR-pTub8-PEPCK-1-4myc-K3R-3’UPRT and 5’UPRT-DHFR-pTub8-PEPCK-1-4myc-K3Q-3’UPRT correspond to second copy expressing vectors where PEPCK-1 lysines K223, K231 and K591 were all sequentially mutated to either arginine or glutamine respectively. Prior to transfection, all PEPCK-1 complementation plasmids were linearised by digestion with NotI-HF and AvrII. Linearised plasmids were co-transfected with 5 μg pSAG1::CAS9-GFP-U6::sgUPRT [59]. Transgenic parasites were selected with pyrimethamine and cloned in 96-well plates. Expression of PEPCK-1 was validated by IFA.

#### KO strains

The KO of ACL (TGME49_223840) and BCKDH-E1a (TGME49_239490) were previously described [13, 14]. To generate the KO of PEPCK-1 (TGME49_289650), a plasmid (pTub-CAT-PEPCK-1-ko) was generated and around 1.5 kb of the 5′ and 3′ flanking regions of PEPCK-1 were amplified using primer pairs 5-6 and 7-8, respectively. The 5′ flanking region was then cloned between KpnI and HindIII restriction sites of the pTub5-CAT and the 3′ flanking region between the BamHI and NotI sites. The plasmid was cut with KpnI and NotI prior to transfection. The KO of FNT-1 (TGME49_209800) was generated as follows: 2gRNA plasmid for the FNT-1 KO was generated as described previously [16] with primers as described in Additional file 14: Table S8. Forward and reverse primers with homology to either the DHFR-TS (p2854-DHFR) selection cassette and to the 5′ and 3′ coding sequence of the gene were generated (KOD polymerase, Novagen). Primers as listed in Additional file 14: Table S8, 10 μg of the 2gRNA plasmid, together with the precipitated KOD PCR product, was transfected. Primers to check for successful integration were used on extracted genomic DNA as shown in Additional file 14: Table S8.

#### Parasite transfection and selection of stable transformants

Parasite transfections were performed by electroporation as previously described [63]. Either mycophenolic acid (MPA, 25 mg/ml) and xanthine (50 mg/ml), pyrimethamine (1 μg/ml) or chloramphenicol (20 μg/ml) were used to select the resistant parasites carrying the HXGPRT, DHFR-TS or CAT cassette, respectively.

#### Antibodies

The antibodies used in this study were previously described as follows: the polyclonal rabbit antibodies used in this study include anti-(α)-GAP45 [64] and α-TgPRF [65]. Monoclonal mouse antibodies include α-TgActin [66], α-myc (9E10), α-Ty (BB2) [67], α-SAG1 (generous gift from Dr. J-F Dubremetz), 5F4 (α-F1B- ATPase, kindly provided by PJ Bradley) [68], α-Atrx1 [69]and α-acetyl-lysine (Cell Signaling Technology). For western blot analyses, secondary horseradish peroxidase (HRP)-conjugated goat α-rabbit or α-mouse antibodies (Sigma) were used. For IFAs, the secondary antibodies Alexa Fluor 488- and Alexa Fluor 594-conjugated goat α-mouse or goat α-rabbit antibodies (Life Technologies) were used.

#### IFAs and confocal microscopy

iΔACS or ΔACL/iΔACS parasites were grown in HFF cells seeded on coverslips for 24 h in the presence or absence of Shld-1. Similarly, stable transgenic parasite lines were grown in HFFs seeded on coverslips. Cells were fixed with 4% paraformaldehyde/0.05% glutaraldehyde (PFA/GA) in phosphate-buffered saline (PBS) for 10 min at room temperature. Fixed cells were then processed as previously described [70]. Confocal images were generated with a Zeiss LSM700 laser scanning confocal microscope using an apochromat × 63/1.4 oil objective. Image stacks were processed with ImageJ and projected using the maximum projection tool.

#### Western blot analyses

iΔACS and ΔACL/iΔACS intracellular parasites were incubated in the presence or absence of Shld-1 for varying durations as indicated. Freshly egressed parasites were lysed in radioimmunoprecipitation assay (RIPA) buffer (150 mM sodium chloride (NaCl), 1% Triton X-100, 0.5% deoxycholate, 0.1% SDS and 50 mM Tris(hydroxymethyl)-aminomethan (TRIS) pH 7.5) for 10 min on ice and mixed with sodium dodecyl sulfate (SDS)-polyacrylamide gel electrophoresis (PAGE) loading buffer under reducing conditions and subjected to sonication. Protein lysates were separated by SDS-PAGE and transferred onto nitrocellulose membranes before blocking in 5% non-fat milk in PBS-0.05% Tween-20 or TRIS-buffered saline with Tween-20 (TBST), followed by incubation of the primary antibody diluted in 5% milk-TBST.

### Transmission electron microscopy

RH and ΔACL/iΔACS parasites were inoculated on confluent HFFs and allowed to grow for 24 h in the presence or absence of Shld-1. Infected host cells were washed with 0.1 M phosphate buffer pH 7.4, then fixed with 2.5% GA in 0.1 M phosphate buffer pH 7.4, scraped and pelleted. Samples were further processed and examined using a Technai 20 electron microscope (FEI Company) as described before [68].

### Transcriptomic analysis

#### Extraction of RNA

RH and ΔACL/iΔACS (following 16 h growth in the absence of Shld-1) or ΔBCKDH parasites were analysed. Biological triplicates of infected HFFs were collected following a rinse with cold PBS and pelleted by centrifugation at (1000*g*, 10 min). Total RNA from the samples was isolated using a hybrid RNA extraction protocol with TRIzol (Life Technologies) and QIAGEN RNeasy Mini Kit. First, sample pellets were lysed in TRIzol followed by the separation of aqueous/organic fractions with the addition of chloroform. RNA from the aqueous phase was precipitated with 70% ethanol, loaded onto RNeasy columns and further processed according to the manufacturer’s instructions.

#### RNA sequencing and data analysis

Isolated RNA was subjected to 100-bp single read sequencing on an Illumina HiSeq 2500 at the iGE3 genomics platform of the University of Geneva (http://www.ige3.unige.ch/genomics-platform.php) (Genomics platform, Institute of Genetics and Genomics iGE3, University of Geneva). Two sets of samples (2 × 3 replicates) were loaded per sequencing lanes of the flow cell. Adapter sequences from the raw reads were trimmed using FASTX-Toolkit (http://hannonlab.cshl.edu/fastx_toolkit/) (phred<20). Following quality control, resulting reads were aligned to the *T. gondii* reference genome (ToxoDB-12) with TopHat/Bowtie2 aligner, and HTSeq-count was used to generate the read counts of the genes [71–74]. edgeR, a Bioconductor package in R (http://www.R-project.org), was implemented to do differential expression analysis. All computations were performed on the Baobab cluster at the University of Geneva. The dataset is available in a data repository [48].

### Proteomic analyses

#### Sample preparation

RH and ΔACL/iΔACS (following 16 h growth in the absence of Shld-1) or ΔBCKDH parasites were analysed. Freshly egressed parasites were lysed in 8 M urea and 50 mM (4-(2-hydroxyethyl)-1-piperazineethanesulfonic acid) (HEPES). Extracted proteins were reduced using 20 mM of dithiothreitol for 1 h at 37 °C before alkylation with 55 mM of iodoacetamide for 45 min at room temperature in the dark. The samples were then diluted using ammonium bicarbonate to obtain a urea concentration of 4 M. Proteins were digested with LysC (Promega) at a ratio of 1:200 during 4 h at 37 °C. The samples were diluted again using ammonium bicarbonate to obtain a urea concentration of 1 M. Proteins were then digested with Trypsin (Promega) at a ratio of 1:200 overnight at 37 °C. Resulting peptides were purified by C18 reverse phase chromatography (Sep-Pak C18, Waters) before drying down. For the comparison of the total proteomes of the ΔACL/iΔACS mutant strain and its parental RH strain, the samples were further fractionated by tip-based strong cation exchange (3M Empore). Briefly, peptides were dissolved in 5% acetonitrile and 1% trifluoracetic acid (TFA) and eluted in 4 fractions (F1: 100 mM ammonium acetate, 20% acetonitrile, 0.5% formic acid; F2: 175 mM ammonium acetate, 20% acetonitrile, 0.5% formic acid; F3: 375 mM ammonium acetate, 20% acetonitrile, 0.5% formic acid; F4: 80% acetonitrile, 5% ammonium hydroxide) before desalting using C18 reverse phase chromatography (Ultra-Micro SpinColumns, Harvard Apparatus). Technical triplicates were performed.

#### Enrichment of acetylated peptides

Dried peptides were dissolved in IP buffer (100 mM NaCl, 1 mM ethylenediaminetetraacetate (EDTA), 20 mM TRIS-HCl, 0.5% NP-40, pH 8). Acetylated peptides were mixed with α-acetyl-lysine antibody immobilised on agarose beads (ImmuneChem-ICP0388) and incubated overnight at 4 °C. Following incubation, the beads were washed twice with IP buffer, once with washing buffer (IP buffer without NP-40) and twice with ice cold ultra-pure water. Elution was performed using 0.1% TFA. Enriched peptides were then dried down. Three technical replicates were prepared from each sample.

#### NanoLC-MS/MS analyses

NanoLC-MS/MS analyses were performed using an Ultimate 3000 RSLCnano coupled to a Q-Exactive Plus (Thermo Scientific). Peptides were sampled on a 300 μm × 5 mm PepMap C18 precolumn and separated on a PepMap 75 μm × 250 mm C18 column (2 μm, Thermo Scientific). The nanoLC methods consisted of 120 or 240 min gradients at a flow rate of 300 nl/min for analysis of respectively enriched acetylated peptides, strong cation exchange fractions and unfractionated total proteome. Spray voltage was set at 1.6 kV, and heated capillary was adjusted to 250–270 °C.

For acetylome analyses, survey full-scan MS spectra (*m/z* 400–1600) were acquired with a resolution of 70,000, with AGC target set to 10^6^ ions (maximum filling time 250 ms) and with lock mass option activated. The 10 most intense ions were fragmented by higher-energy collisional dissociation (nce = 30) with a resolution of 17,500, with AGC target set to 10^6^ ions (maximum filling time 250 ms). Analytical triplicates were acquired for the comparison of acetylomes between ΔBCKDH mutant strain and its parental RH strain.

For total proteome analyses, survey full-scan MS spectra (*m/z* 400–1600) were acquired with a resolution of 70,000, with AGC target set to 10^6^ ions (maximum filling time 200 ms) and with lock mass option activated. The 10 most intense ions were fragmented by higher-energy collisional dissociation (nce = 30) with a resolution of 17,500, with AGC target set to 10^5^ ions (maximum filling time 50 ms). MS and tandem MS data were acquired using the Xcalibur software (Thermo Scientific). Analytical quadruplicates were acquired for comparison of total proteomes of ΔBCKDH mutant strain and its parental RH strain.

Raw and processed MS data have been deposited in a data repository [28, 75].

#### Peptide, protein and acetylated site identifications

RAW files were processed using MaxQuant [76] version 1.5.8.3. Spectra were searched against the *T. gondii* database (ME49 taxonomy, version 30 downloaded from ToxoDB [77], the Swiss-Prot database (*Homo sapiens* taxonomy, 2017-08-24 version), the frequently observed contaminants database embedded in MaxQuant and the corresponding reverse databases. Trypsin was chosen as the enzyme, and two or three missed cleavages were allowed for respectively total proteome and acetylome analyses. Precursor and fragment mass error tolerances were set at their default values. Peptide modifications allowed during the search were carbamidomethyl (C, fixed), acetyl (protein N-term, variable), oxidation (M, variable) for total proteome analyses, and acetyl (K, variable) was added for acetylome analyses. Minimum number of peptides, razor + unique peptides and unique peptides were all set to 1. Maximum false discovery rates were set to 0.01 at PSM and site levels for acetylome analyses and at 0.01 at PSM, protein and site levels for total proteome analyses. The match between runs option was selected. For total proteome analyses, label-free protein quantitation (LFQ) was performed with a minimum ratio count of 2. For acetylome analyses, only acetylated sites with a probability above 0.9 were considered.

#### Statistical analyses

Statistical analyses were performed using ProStaR [78]. Peptides and proteins identified in the reverse and contaminant databases or matching to human sequences were discarded. For the comparison of acetylomes between ΔBCKDH mutant strain and its parental RH strain, intensities of the acetylated sites were deduced through averaging the values extracted from the analytical triplicates. For differential analysis of acetylomes, acetylated sites were split into several lanes when they were identified on peptides bearing various numbers of acetylated sites to allow the quantification of the different peptidoforms. Only proteins and acetylated sites detected in at least 3 replicates of one condition were conserved. After log2 transformation, protein LFQ intensities and acetylated site intensities were median normalised before missing value imputation (replacing missing values by the first percentile value of each column). Statistical testing was conducted using *limma*. Differentially abundant proteins and acetylated sites were sorted out using the following cut-offs: log2(fold change) ≥ 1 or ≤ − 1 and *p* value < 0.01, allowing to reach an FDR inferior to 5% according to the Benjamini-Hochberg estimator.

#### GO enrichment analyses

GO enrichment analyses of transcripts and proteins was carried out using the tool for enrichment in biological processes on ToxoDB (https://toxodb.org/) against the total *T. gondii* database with a *p* value cut-off of 0.05 (limited to GO slim terms function). GO enrichment analysis of the acetylome was performed using the R package, topGO (version 2.38.1) [29]. Enrichment factors were calculated as the ratio of the significant genes assigned to the GO to the expected number of assigned genes defined by topGO. Enrichments in biological processes were determined in comparison with either the total *T. gondii* genome/proteome (*p* value cut-off, 0.001) or against the subset of identified acetylated proteins (*p* value cut-off, 0.05).

### Metabolomic analyses

#### Metabolomics sample preparation

Triplicates of RH, iΔACS and ΔACL/iΔACS intracellular parasites were incubated in the absence of Shld-1 for 16 h. Freshly egressing parasites were extracted through repeated syringe lysis (3×, 28G), purified from host cell material through filtration (3 μm pore size, Millipore/Merck) and pelleted by centrifugation (2800 *g*, 20 min, 4 °C). Pellets were washed with ice-cold PBS (3×), and metabolites were extracted as outlined below.

#### Untargeted LC-MS analysis

Metabolites were extracted in 80% acetonitrile and 20% ultra-pure water through vigorous vortexing. An aliquot (10 μl) of each sample was combined to generate a pooled biological quality control (PBQC) sample, which was used to monitor downstream sample stability and analytical reproducibility. Metabolomics analysis was performed by LC-MS, using hydrophilic interaction liquid chromatography (ZIC-pHILIC, Merck) and high-resolution MS (Q-Exactive Orbitrap, Thermo Fisher) as previously described [79]. Sample injections within the experiment were randomised to avoid any impact of systematic instrument drift on metabolite signals. Retention times for 250 authentic standards were checked manually to aid metabolite identification.

Identification and quantification of metabolites were performed using the IDEOM workflow [80]. Over 850 metabolites were putatively identified based on accurate mass and standard retention time (where available) (confidence score 1 in Additional file 2: Table S1), or predicted retention time (confidence score 2 in Additional file 2: Table S1) [81]. Metabolite abundance was determined by LC-MS peak height and was normalised to the average for wild-type (RH) samples. Statistical analyses utilised Welch’s *t*-test (*α* = 0.05), and pathway analysis used KEGG annotations (Microsoft Excel). Stable isotope labelling was analysed using mzMatch-ISO [82] to extract all isotopologues of all putatively identified metabolites based on retention time and accurate mass. The complete dataset is available in a data repository [24].

#### FA profiling by GC-MS

RH and ΔACL/iΔACS intracellular parasites were incubated in the absence of Shld-1 for 16 h and simultaneously labelled with either 10 mM U-^13^C-glucose or 2 mM U-^13^C-acetate (Cambridge Isotope Laboratories). Per sample, 10^8^ parasites were isolated and purified as described above. FAs were extracted, derivatised and analysed as described previously [16]. Fatty acid methyl esters (FAMEs) were identified based on retention times and the library integrated in Xcalibur (Thermo Fisher Scientific). The abundance was determined based on the peak intensity relative to the cholesterol signal intensity in each sample. Abundance of the C14:0 and C26:1-FAME mass isotopologues (*m/z* 242–256; *m/z* 376–390) was determined using Xcalibur (Thermo Fisher Scientific) and OpenChrom software. The extent of ^13^C-labelling was determined using the Excel (Microsoft) software following correction for the occurrence of natural isotopes as described by Zamboni et al. [83]. Abundance data represent the average of 6 biological replicates, and labelling data represents the average of 3 biological replicates. Statistical significance of differences in labelling and abundance were assessed by *t*-test. Standard deviation and *p* values are indicated in the figure.

#### Polar metabolite profiling in PEPCK-1 acetylation mimetics

Intracellular parasites were cultured in the presence of equimolar (8 mM) levels of natural-abundance glucose and U-^13^C-glutamine (Cambridge Isotope Laboratories) for 24 h. Freshly egressing parasites (10^8^ cells) were purified and harvested and metabolites extracted as described above. Polar metabolite extracts were prepared and analysed as described previously [16]. Aspartate and glucose-6-phosphate were identified based on their retention time and ion spectrum of authentic standards. The signal for mass isotopologues of major ions of aspartate (*m/z* 232–235) and glucose-6-P (*m/z* 471–475) was determined using Xcalibur (Thermo Fisher Scientific) and OpenChrom software. The extent of ^13^C-labelling in these ions was determined using the Excel (Microsoft) software following correction for the occurrence of natural isotopes as described by Zamboni et al. [83]. Displayed data represent the average of 3 biological replicates. The standard deviation is indicated.

#### Profiling of intracellular and secreted metabolites in ΔBCKDH and ΔFNT-1 cells

Freshly egressing tachyzoites (10^8^ parasites) of each strain were harvested and purified as described above and incubated (3 h, 37 °C) in 1.5 ml of glucose-free DMEM (Gibco) supplemented with 12 mM U-^13^C-glucose (Cambridge Isotope Laboratories). Subsequently, parasites were rapidly chilled and pelleted by centrifugation (6000 *g*, 10 min, 4 °C). Culture medium was taken from the supernatant and stored at − 80 °C. Cell pellets were washed 3× with PBS and intracellular metabolites extracted as described previously [16] with the polar phase (300 μl) containing 5 μM scyllo-inositol (Sigma-Aldrich/Merck) as an internal standard. Twenty microlitres of cell culture medium and polar metabolite extracts was dried down in MS vial inserts using a centrifugal evaporator. The dried medium samples and cell extracts were derivatised using methoxyamine hydrochloride (Sigma-Aldrich/Merck) and *N*,*O*-bis(trimethylsilyl)trifluoroacetamide trimethylchlorosilane (Sigma-Aldrich/Merck) as described previously [16]. Metabolites were identified based on the retention time and ion spectrum of authentic standards and were quantified by integrating the area under the peak in the total ion chromatogram (TIC) using Xcalibur (Thermo Fisher Scientific) and OpenChrom software and comparing their abundance to that of the internal standard scyllo-inositol (intracellular metabolites) or to the abundance of valine (medium samples). The relative abundance of metabolites was determined using the Excel (Microsoft) software. Displayed data represent the average of 3–5 biological replicates. Standard deviation between replicates is indicated by the error bars, and statistically significant differences were tested by *t*-test and are as indicated in the figure.

## Supplementary information

**Additional file 1 **: **Figure S1.** Generation of ΔACL and iΔACS parasites. PDF image showing a schematic representations of the strategies used to introduce a DDmyc destabilisation domain at the ATG of the locus of *acs* generating iΔACS (**a**) and to delete *acl* (ΔACL) using double homologous recombination (**b**), scheme adapted from [14]. PCRs were performed on genomic DNA extracted from clones and using primers listed in Additional file 14: Table S8, confirming correct integration of the constructs (**c**). Abbreviations: GOI: gene of interest; FS: flanking sequence; gRNA: guide RNA; ACL: ATP-citrate lyase; ACS: acetyl-CoA synthetase; HXGPRT: hypoxanthine-xanthine-guanine phosphoribosyl transferase; DD: destabilization domain; PCR: polymerase chain reaction; DNA: deoxyribonucleic acid.

**Additional file 2 **: **Table S1.** ΔACL/iΔACS metabolome. Excel spreadheet summarising the metabolomic data set from the LC-MS analysis. Metabolites which are significantly decreased or increased in ΔACL parasites, iΔACS parasites and ΔACL/iΔACS parasites are highlighted. Abbreviations: ACL: ATP-citrate lyase; ACS: acetyl-CoA synthetase; LC-MS: liquid chromatography-mass spectrometry.

**Additional file 3 **: **Figure S2.** Alterations in metabolite abundances and FA labelling in ΔACL/iΔACS parasites. PDF image providing an (**a**) overview of metabolites which were significantly altered in their abundance in ΔACL/iΔACS parasites compared to RH (RH abundance = 1). Metabolites were grouped according to their class. Error bars indicate the standard deviation between triplicates. All displayed metabolites were found to be significantly de- or increased by t-test (*p*<0.05). (**b**) Fractional ^13^C-labelling from U-^13^C-glucose (U-^13^C-Glc) and U-^13^C-acetate (U-^13^C-Ac) in myristate (FA C14:0) and FA C26:1 was measured by GC-MS following labelling of RH and ΔACL/iΔACS parasites for 16 hours during simultaneous ACS depletion. Error bars represent the standard deviation between replicates. Statistical significance was test using a t-test and is as indicated (* - p<0.05; *** - *p*<0.0001). Abbreviations: ACL: ATP-citrate lyase; ACS: acetyl-CoA synthetase; FA: fatty acid; GC-MS: gas chromatography-mass spectrometry; Glc: glucose; Ac: acetate.

**Additional file 4 **: **Figure S3.** Stable isotope labelling to elucidate the consequences of the loss of ACL and/or ACS. PDF image showing (**a**) Fractional ^13^C-labelling from U-^13^C-glucose in glycolysis and TCA-cycle intermediates comparing RH, ΔACL iΔACS or ΔACL/iΔACS parasites. The inducible knock-down parasites were grown in the absence of Shield-1 for 16 hours prior to analysis. (**b**) Fractional ^13^C-labelling from U-^13^C-acetate in glycolysis and TCA-cycle intermediates. (**c-f**) Mass isotopologue abundance of selected metabolites following labelling with U-^13^C-acetate: citrate (**c**), ceramide Cer(36:1) (**d**), phosphatidylserine PS(36:2) (**e**) and PS(38:2) (**f**). Legend in c-e as in f. Abbreviations: TCA: tricarboxylic acid; ACL: ATP-citrate lyase; ACS: acetyl-CoA synthetase.

**Additional file 5 **: **Figure S4.** Acetylome analysis of ΔACL/iΔACS and ΔBCKDH parasites. PDF image of the (**a**) Western blot analysis representing the total lysine acetylation profile of ΔACL/iΔACS parasites. ΔACL/iΔACS parasites were cultured in the absence of Shield-1 for 16 hours prior to harvest and analysis. The blot was probed with α-acetyl-lysine antibody for the total lysate and α-actin as loading control. (**b**) Western blot analysis representing the total lysine acetylation profile of ΔBCKDH parasites. The blot was probed with α-acetyl-lysine antibody for the total lysate and α-actin as loading control. (**c**) The localisation of all differentially acetylated proteins in ΔACL/iΔACS parasites is shown. Sub-cellular localisation of differentially acetylated proteins and enrichment was determined using the hyperplexed Localisation of Organelle Proteins by Isotopic Tagging (hyperLOPIT) data available under https://proteome.shinyapps.io/toxolopittzex/. (**d**) The localisation of differentially acetylated proteins in ΔBCKDH parasites was determined as above and is displayed. (**e**) Proteins identified as differentially acetylated in ΔACL/iΔACS parasites were analysed using GO-enrichment R-package topGO to identify enrichment in biological processes using the relatively small subset of acetylated proteins as background. Enrichment was 1.3- (GO:0044267 – cellular protein metabolic process) and 1.9-fold (GO:0051276 – chromosome organization). Statistically significant enrichment was assessed by Fisher’s exact test (*p*-value <0.05). (**f**) Proteins identified as differentially acetylated in ΔBCKDH parasites were analysed using GO-enrichment R-package topGO to identify enrichment in biological processes using the relatively small subset of acetylated proteins as background. Enrichment ranged from 2.3- (GO:0072350 – tricarboxylic acid metabolic process) to 1.6-fold (all other enriched biological processes). Statistically significant enrichment was assessed by Fisher’s exact test (p-value <0.05). Abbreviations: BCKDH: branched-chain α-keto acid dehydrogenase-complex; ACL: ATP-citrate lyase; ACS: acetyl-CoA synthetase; PM: plasma membrane; IMC: inner membrane complex; ER: endoplasmic reticulum.

**Additional file 6 **: **Table S2.** ΔACL/iΔACS acetytlome. Excel spreadheet summarising the acetylome data set from the nanoLC-MS/MS analysis. Sites and proteins which are significantly hypo- or hyper-acetylated in ΔACL/iΔACS parasites are highlighted. Abbreviations: ACL: ATP-citrate lyase; ACS: acetyl-CoA synthetase; LC-MS/MS: liquid chromatography-tandem mass spectrometry.

**Additional file 7 **: **Table S3.** ΔBCKDH acetytlome. Excel spreadheet summarising the acetylome data set from the nanoLC-MS/MS analysis. Sites and proteins which are significantly hypo- or hyper-acetylated in ΔBCKDH parasites are highlighted. Abbreviations: BCKDH: branched-chain α-keto acid dehydrogenase-complex; LC-MS/MS: liquid chromatography-tandem mass spectrometry.

**Additional file 8 **: **Figure S5.** Generation and analysis of PEPCK-1-3Ty knock-in, ΔPEPCK-1 strains and PEPCK-1 acetylation mimetics in *T. gondii*. PDF file showing the (**a**) schematic of the putative mechanism of gluconeogenesis-activation through PEPCK-1 de-acetylation. Gluconeogenesis is inactive in wild-type parasites (RH) under glucose replete conditions (left panel), during which PEPCK is acetylated in position K223. Inactivity of the gluconeogenesis pathway if graphically represented by the faint dashed arrow and PEPCK-1 acetylation is graphically represented by an orange circle. We identified PEPCK-1 to be hypo-acetylated in ΔBCKDH parasites (right panel) and a previous study highlighted the constitutive activation of gluconeogenesis in parasites lacking BCKDH [13]. Activation of gluconeogenesis is represented by the thick arrow. These findings prompted us to hypothesize that PEPCK-1 acetylation in lysine 223 may be responsible for the activation of the pathway and to assess the role of gluconeogenesis in parasites lacking BCKDH. (**b**) Schematic representation of the *pepck*-*1* locus. Two alternative translational starts are represented by inverted triangles (blue) while the translational stop is represented by a red triangle. (**c**) Secondary expression of the long isoform of PEPCK-1 (PEPCK-1-3Ty-L, mitochondrial) and the short PEPCK-1 (PEPCK-1-3Ty-S, cytosolic). Expression of the second PEPCK-1 copy was detected using α-Ty (green) while α-gliding associated protein 45 (α-GAP45, red) was used as a pellicle marker. (**d**) Graphical representation of the knock-in strategy used to introduce a 3Ty tag at the C-terminus of the endogenous *pepck-1* locus in RH parasites. (**e**) PCRs performed on genomic DNA extracted from clones showing correct integration of the construct. (f) Schematic representation of the double homologous recombination strategy used to KO the endogenous *pepck-1* locus in RH and ΔBCKDH parasites. (g) PCRs performed on genomic DNA using primers listed in Additional file 14: Table S8 show correct integration of the construct. (h) Expression of the acetylation mimetics of PEPCK-1 was tested by IFA using an α-Ty antibody. (i) *pepck*-1 was replaced in ΔBCKDH parasites by a *cat* resistance cassette as shown in panel f. PCRs were performed on genomic DNA extracted from clones showing correct integration of the construct. (j) Plaque assays of RH, ΔBCKDH, ΔPEPCK-1 or double KO ΔBCKDH/ΔPEPCK-1 parasites. (k,l) Intracellular growth assays at 24 hours post infection of RH, ΔBCKDH, ΔPEPCK-1 and ΔBCKDH/ΔPEPCK-1 parasites in complete medium (k) or in medium lacking glucose (l). Error bars represent the standard deviation between 3 independent infections. Per infection >100 vacuoles were counted. Throughout the figure PEPCK refers to PEPCK-1, the active enzyme in tachyzoites [42]. Abbreviations: GNG: gluconeogenesis; PEP: phosphoenolpyruvate; Gln: glutamine; Cit: citrate; OAA: oxaloacetate; PM: plasma membrane; PEPCK: phosphoenolpyruvate carboxykinase-1; BCKDH: branched-chain α-keto acid dehydrogenase-complex; DHFR-TS: dihydrofolate reductase-thymidylate synthase; CAT: chloramphenicol acetyltransferase; FS: flanking sequence; PCR: polymerase chain reaction; DNA: deoxyribonucleic acid.

**Additional file 9 **: **Table S4.** ΔACL/iΔACS transcriptome. Excel spreadheet summarising the transcriptome data set from the RNA-seq analysis. Transcripts which are significantly up- or downregulated in ΔACL/iΔACS parasites are highlighted. Abbreviations: ACL: ATP-citrate lyase; ACS: acetyl-CoA synthetase.

**Additional file 10 **: **Table S5.** ΔACL/iΔACS proteome. Excel spreadheet summarising the proteome data set from the nano-LC-MS/MS analysis. Proteins which are significantly up- or downregulated in ΔACL/iΔACS parasites are highlighted. Abbreviations: ACL: ATP-citrate lyase; ACS: acetyl-CoA synthetase; LC-MS/MS: liquid chromatography-tandem mass spectrometry.

**Additional file 11 **: **Table S6.** ΔBCKDH transcriptome. Excel spreadheet summarising the transcriptome data set from the RNA-seq analysis. Transcripts which are significantly up- or downregulated in ΔBCKDH parasites are highlighted. Abbreviations: BCKDH: branched-chain α-keto acid dehydrogenase-complex.

**Additional file 12 **: **Table S7.** ΔBCKDH proteome. Excel spreadheet summarising the proteome data set from the nano-LC-MS/MS analysis. Proteins which are significantly up- or downregulated in ΔBCKDH parasites are highlighted. Abbreviations: BCKDH: branched-chain α-keto acid dehydrogenase-complex; LC-MS/MS: liquid chromatography-tandem mass spectrometry.

**Additional file 13 **: **Figure S6.** Generation of parasites lacking BCKDH, FNT-1 or both. PDF image displaying the (**a**) schematic representation of the strategy to deplete the *fnt-1* locus and replace it with a *dhfr-ts* resistance cassette. (**b**) PCRs were performed on genomic DNA extracted from clones and using primers listed in Additional file 14: Table S8, confirming correct integration of the constructs. (**c**) Table summarising the growth defect in the various strains described throughout the manuscript. Abbreviations: BCKDH: branched-chain α-keto acid dehydrogenase-complex; FS: flanking sequence; DHFR-TS: dihydrofolate reductase-thymidylate synthase; FNT: formate/nitrite transporter; PCR: polymerase chain reaction; DNA: deoxyribonucleic acid; ACL: ATP-citrate lyase; ACS: acetyl-CoA synthetase; BCKDH: branched-chain α-keto acid dehydrogenase-complex; PEPCK: phosphoenolpyruvate carboxykinase-1.

**Additional file 14 **: **Table S8.** Primers. Excel spreadsheet providing an overview of the primers used in this publication including their sequences.

## Data Availability

All relevant data are enclosed in the manuscript and/or deposited online as outlined here. Raw and processed proteomic MS data have been deposited with the ProteomeXchange Consortium via the PRIDE partner repository, accession number PXD016133 [28]. Raw sequence read transcriptomic have been deposited in the European Nucleotide Archive, accession number PRJEB36162 [48]. Metabolomics MS data and search results have been deposited with the Metabolomics Workbench Consortium, accession number/Project ID: PR000885 [24].
