# Supplementary figures and images for: Multi-omics analysis delineates the distinct functions of sub-cellular acetyl-CoA pools in Toxoplasma gondii
Source: BMC Biol. 2020 Jun 16;18:67. doi: 10.1186/s12915-020-00791-7 (PMC7296777; doi:10.1186/s12915-020-00791-7)

Additional file 1: Figure S1

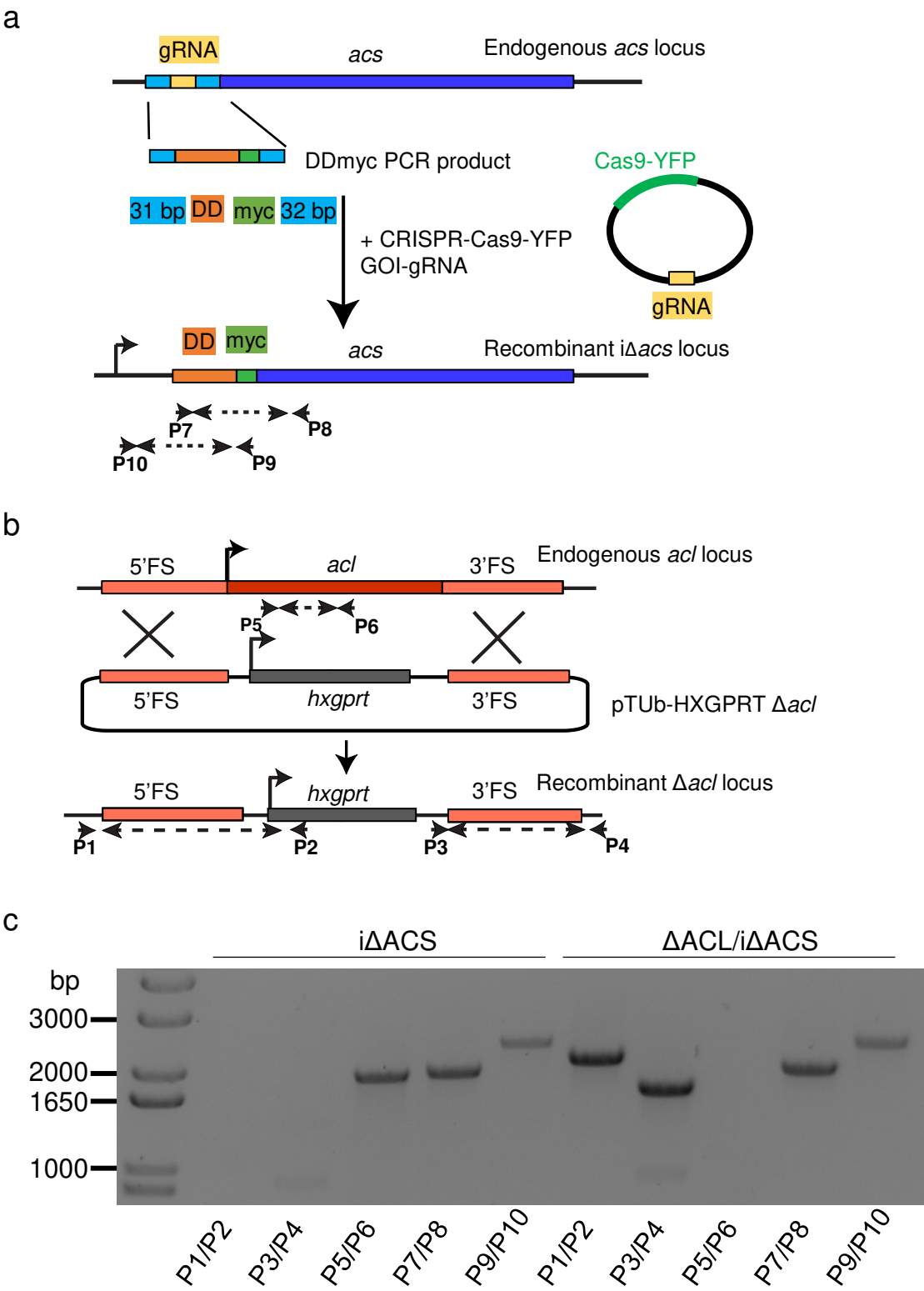

Supplement: Supplementary file 1 — Additional file 1 : Figure S1. Generation of ΔACL and iΔACS parasites. PDF image showing a schematic representations of the strategies used to introduce a DDmyc destabilisation domain at the ATG of the locus of acs generating iΔACS (a) and to delete acl (ΔACL) using double homologous recombination (b), scheme adapted from [14]. PCRs were performed on genomic DNA extracted from clones and using primers listed in Additional file 14: Table S8, confirming correct integration of the constructs (c). Abbreviations: GOI: gene of interest; FS: flanking sequence; gRNA: guide RNA; ACL: ATP-citrate lyase; ACS: acetyl-CoA synthetase; HXGPRT: hypoxanthine-xanthine-guanine phosphoribosyl transferase; DD: destabilization domain; PCR: polymerase chain reaction; DNA: deoxyribonucleic acid. [file 12915_2020_791_MOESM1_ESM.pdf]

Additional file 3: Figure S2

a

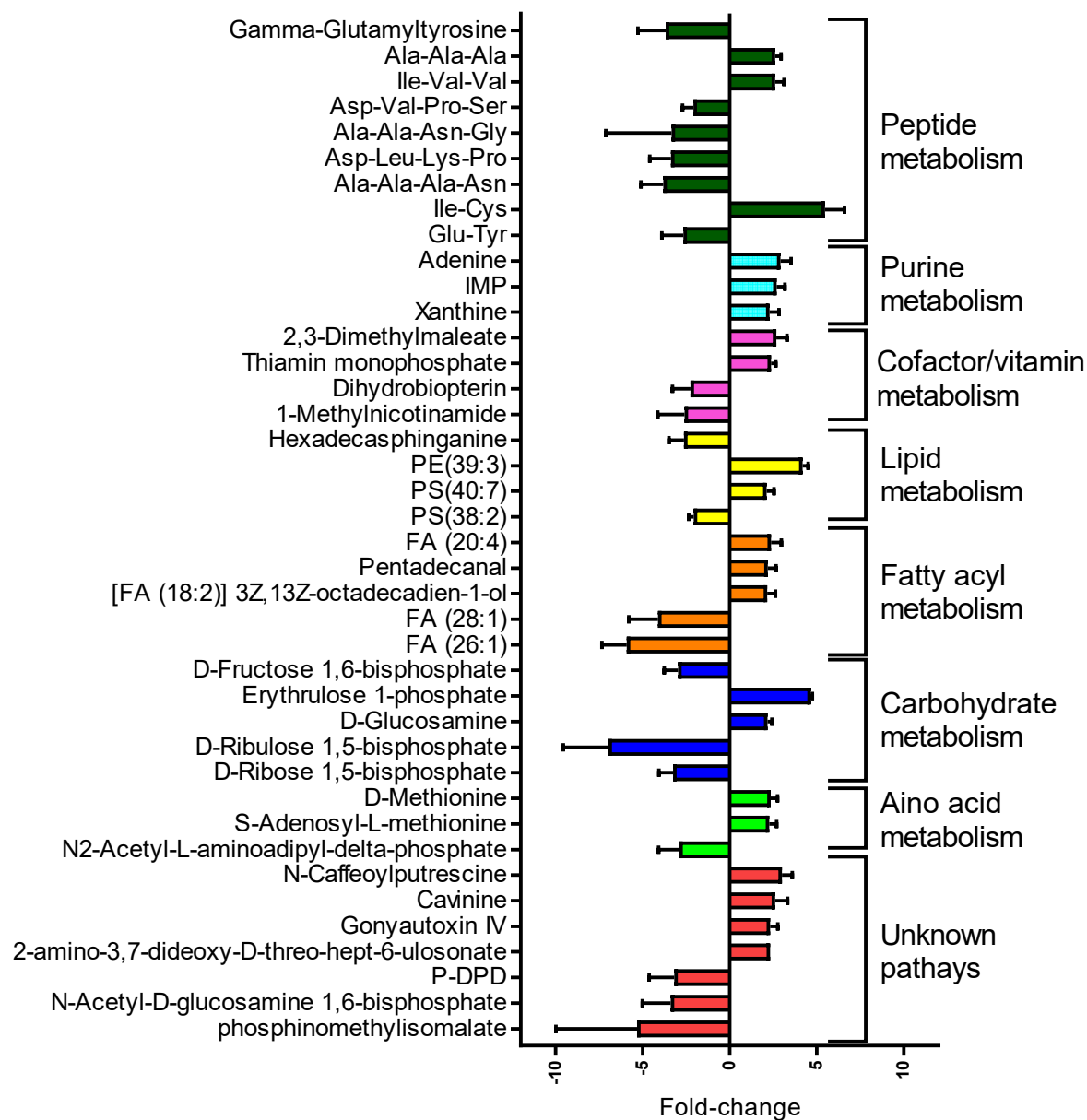

b

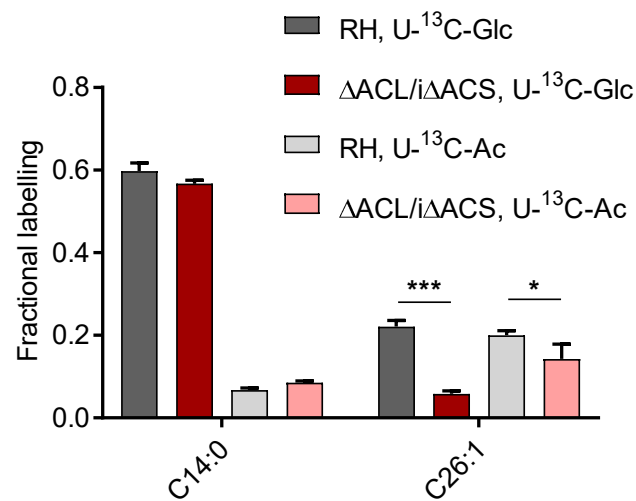

Supplement: Supplementary file 3 — Additional file 3 : Figure S2. Alterations in metabolite abundances and FA labelling in ΔACL/iΔACS parasites. PDF image providing an (a) overview of metabolites which were significantly altered in their abundance in ΔACL/iΔACS parasites compared to RH (RH abundance = 1). Metabolites were grouped according to their class. Error bars indicate the standard deviation between triplicates. All displayed metabolites were found to be significantly de- or increased by t-test (p<0.05). (b) Fractional 13C-labelling from U-13C-glucose (U-13C-Glc) and U-13C-acetate (U-13C-Ac) in myristate (FA C14:0) and FA C26:1 was measured by GC-MS following labelling of RH and ΔACL/iΔACS parasites for 16 hours during simultaneous ACS depletion. Error bars represent the standard deviation between replicates. Statistical significance was test using a t-test and is as indicated (* - p<0.05; *** - p<0.0001). Abbreviations: ACL: ATP-citrate lyase; ACS: acetyl-CoA synthetase; FA: fatty acid; GC-MS: gas chromatography-mass spectrometry; Glc: glucose; Ac: acetate. [file 12915_2020_791_MOESM3_ESM.pdf]

Additional file 4: Figure S3

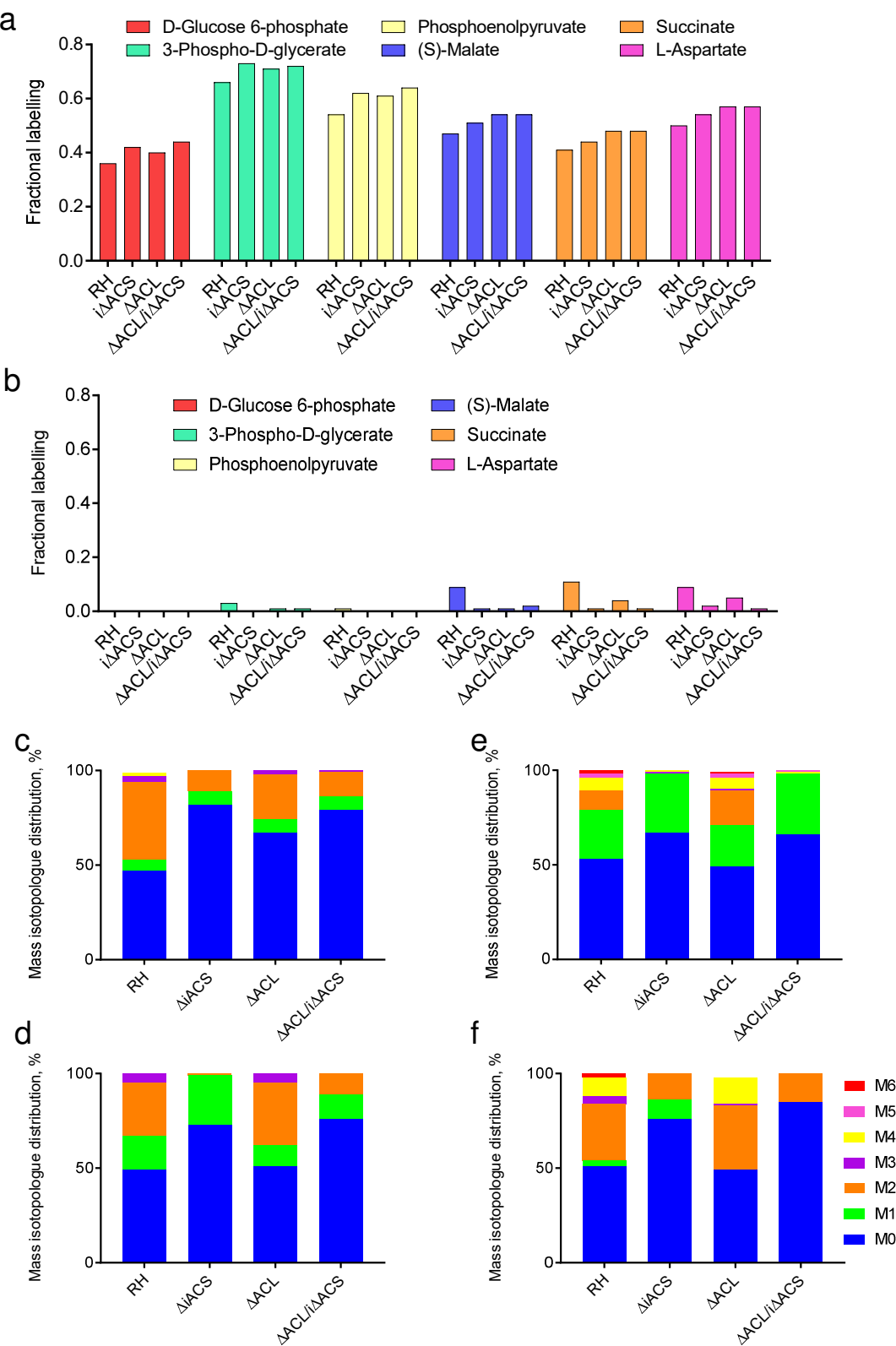

Supplement: Supplementary file 4 — Additional file 4 : Figure S3. Stable isotope labelling to elucidate the consequences of the loss of ACL and/or ACS. PDF image showing (a) Fractional 13C-labelling from U-13C-glucose in glycolysis and TCA-cycle intermediates comparing RH, ΔACL iΔACS or ΔACL/iΔACS parasites. The inducible knock-down parasites were grown in the absence of Shield-1 for 16 hours prior to analysis. (b) Fractional 13C-labelling from U-13C-acetate in glycolysis and TCA-cycle intermediates. (c-f) Mass isotopologue abundance of selected metabolites following labelling with U-13C-acetate: citrate (c), ceramide Cer(36:1) (d), phosphatidylserine PS(36:2) (e) and PS(38:2) (f). Legend in c-e as in f. Abbreviations: TCA: tricarboxylic acid; ACL: ATP-citrate lyase; ACS: acetyl-CoA synthetase. [file 12915_2020_791_MOESM4_ESM.pdf]

Additional file 5: Figure S4

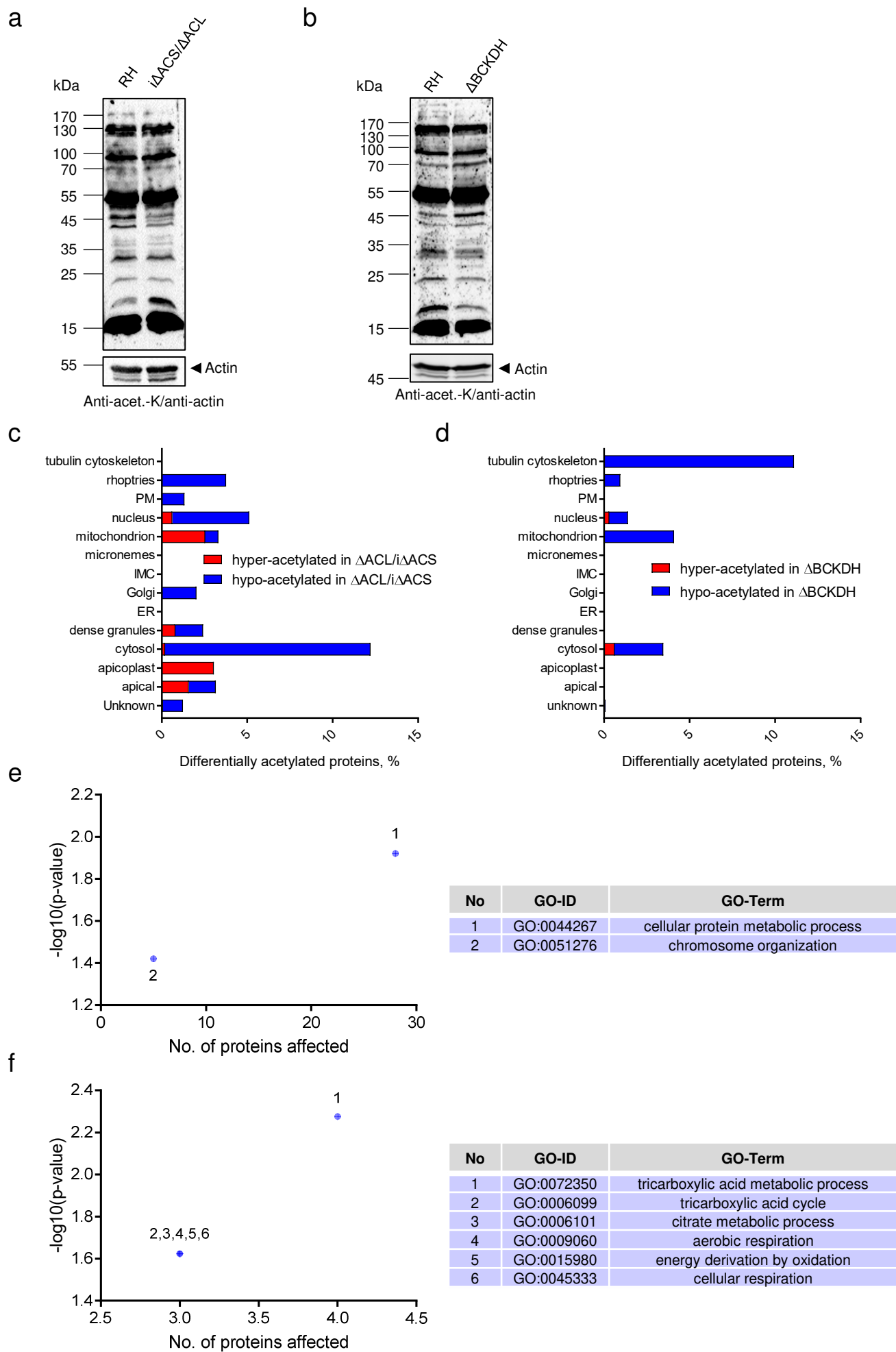

Supplement: Supplementary file 5 — Additional file 5 : Figure S4. Acetylome analysis of ΔACL/iΔACS and ΔBCKDH parasites. PDF image of the (a) Western blot analysis representing the total lysine acetylation profile of ΔACL/iΔACS parasites. ΔACL/iΔACS parasites were cultured in the absence of Shield-1 for 16 hours prior to harvest and analysis. The blot was probed with α-acetyl-lysine antibody for the total lysate and α-actin as loading control. (b) Western blot analysis representing the total lysine acetylation profile of ΔBCKDH parasites. The blot was probed with α-acetyl-lysine antibody for the total lysate and α-actin as loading control. (c) The localisation of all differentially acetylated proteins in ΔACL/iΔACS parasites is shown. Sub-cellular localisation of differentially acetylated proteins and enrichment was determined using the hyperplexed Localisation of Organelle Proteins by Isotopic Tagging (hyperLOPIT) data available under https://proteome.shinyapps.io/toxolopittzex/. (d) The localisation of differentially acetylated proteins in ΔBCKDH parasites was determined as above and is displayed. (e) Proteins identified as differentially acetylated in ΔACL/iΔACS parasites were analysed using GO-enrichment R-package topGO to identify enrichment in biological processes using the relatively small subset of acetylated proteins as background. Enrichment was 1.3- (GO:0044267 – cellular protein metabolic process) and 1.9-fold (GO:0051276 – chromosome organization). Statistically significant enrichment was assessed by Fisher’s exact test (p-value <0.05). (f) Proteins identified as differentially acetylated in ΔBCKDH parasites were analysed using GO-enrichment R-package topGO to identify enrichment in biological processes using the relatively small subset of acetylated proteins as background. Enrichment ranged from 2.3- (GO:0072350 – tricarboxylic acid metabolic process) to 1.6-fold (all other enriched biological processes). Statistically significant enrichment was assessed by Fisher’s exact test (p-value [file 12915_2020_791_MOESM5_ESM.pdf]

Additional file 8: Figure S5

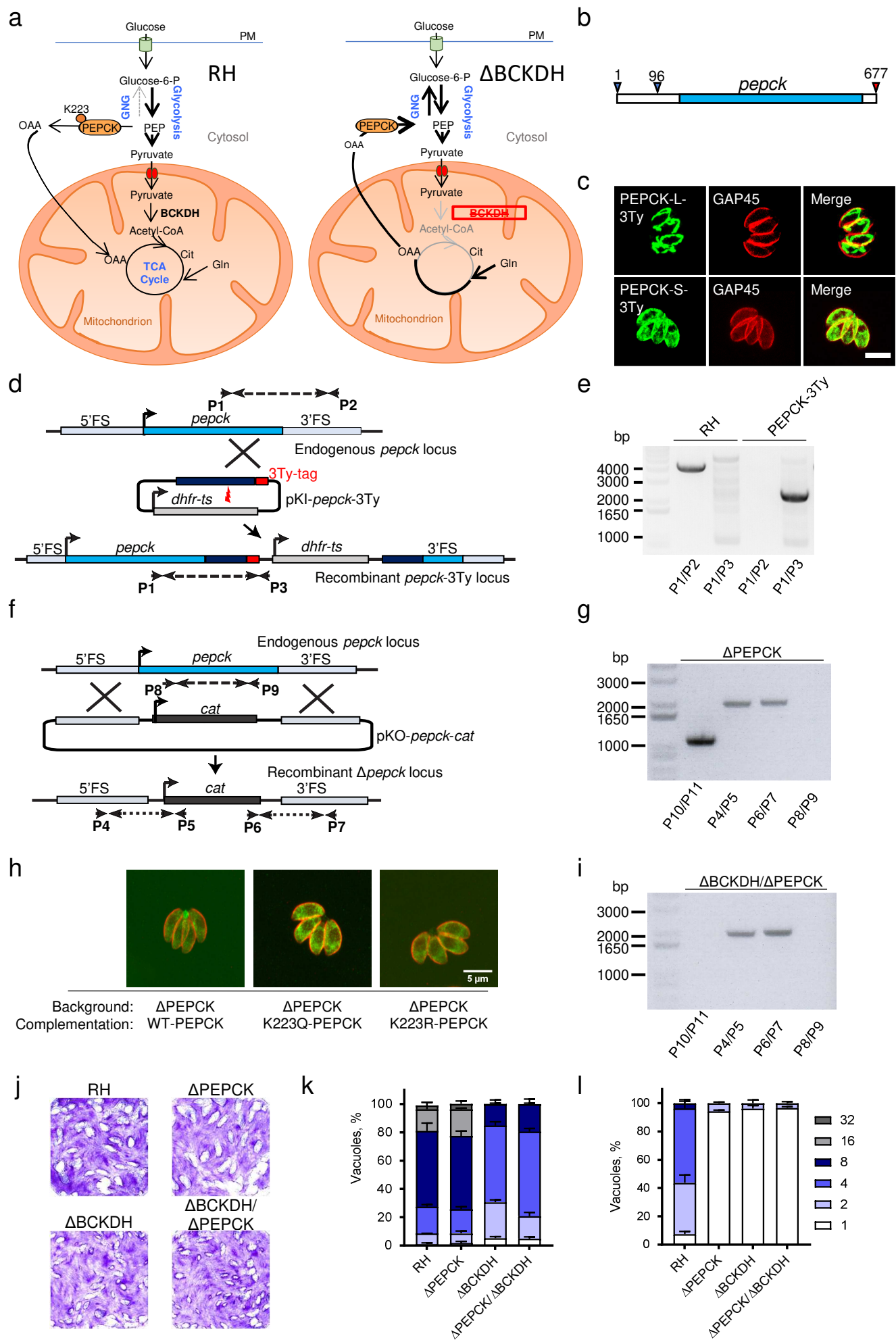

Supplement: Supplementary file 8 — Additional file 8 : Figure S5. Generation and analysis of PEPCK-1-3Ty knock-in, ΔPEPCK-1 strains and PEPCK-1 acetylation mimetics in T. gondii. PDF file showing the (a) schematic of the putative mechanism of gluconeogenesis-activation through PEPCK-1 de-acetylation. Gluconeogenesis is inactive in wild-type parasites (RH) under glucose replete conditions (left panel), during which PEPCK is acetylated in position K223. Inactivity of the gluconeogenesis pathway if graphically represented by the faint dashed arrow and PEPCK-1 acetylation is graphically represented by an orange circle. We identified PEPCK-1 to be hypo-acetylated in ΔBCKDH parasites (right panel) and a previous study highlighted the constitutive activation of gluconeogenesis in parasites lacking BCKDH [13]. Activation of gluconeogenesis is represented by the thick arrow. These findings prompted us to hypothesize that PEPCK-1 acetylation in lysine 223 may be responsible for the activation of the pathway and to assess the role of gluconeogenesis in parasites lacking BCKDH. (b) Schematic representation of the pepck-1 locus. Two alternative translational starts are represented by inverted triangles (blue) while the translational stop is represented by a red triangle. (c) Secondary expression of the long isoform of PEPCK-1 (PEPCK-1-3Ty-L, mitochondrial) and the short PEPCK-1 (PEPCK-1-3Ty-S, cytosolic). Expression of the second PEPCK-1 copy was detected using α-Ty (green) while α-gliding associated protein 45 (α-GAP45, red) was used as a pellicle marker. (d) Graphical representation of the knock-in strategy used to introduce a 3Ty tag at the C-terminus of the endogenous pepck-1 locus in RH parasites. (e) PCRs performed on genomic DNA extracted from clones showing correct integration of the construct. (f) Schematic representation of the double homologous recombination strategy used to KO the endogenous pepck-1 locus in RH and ΔBCKDH parasites. (g) PCRs performed on genomic DNA using primers listed in Additional [file 12915_2020_791_MOESM8_ESM.pdf]
