# Supplementary material for: Multi-omics analysis delineates the distinct functions of sub-cellular acetyl-CoA pools in Toxoplasma gondii
Source: BMC Biol. 2020 Jun 16;18:67. doi: 10.1186/s12915-020-00791-7 (PMC7296777; doi:10.1186/s12915-020-00791-7)

Additional file 13: Figure S6

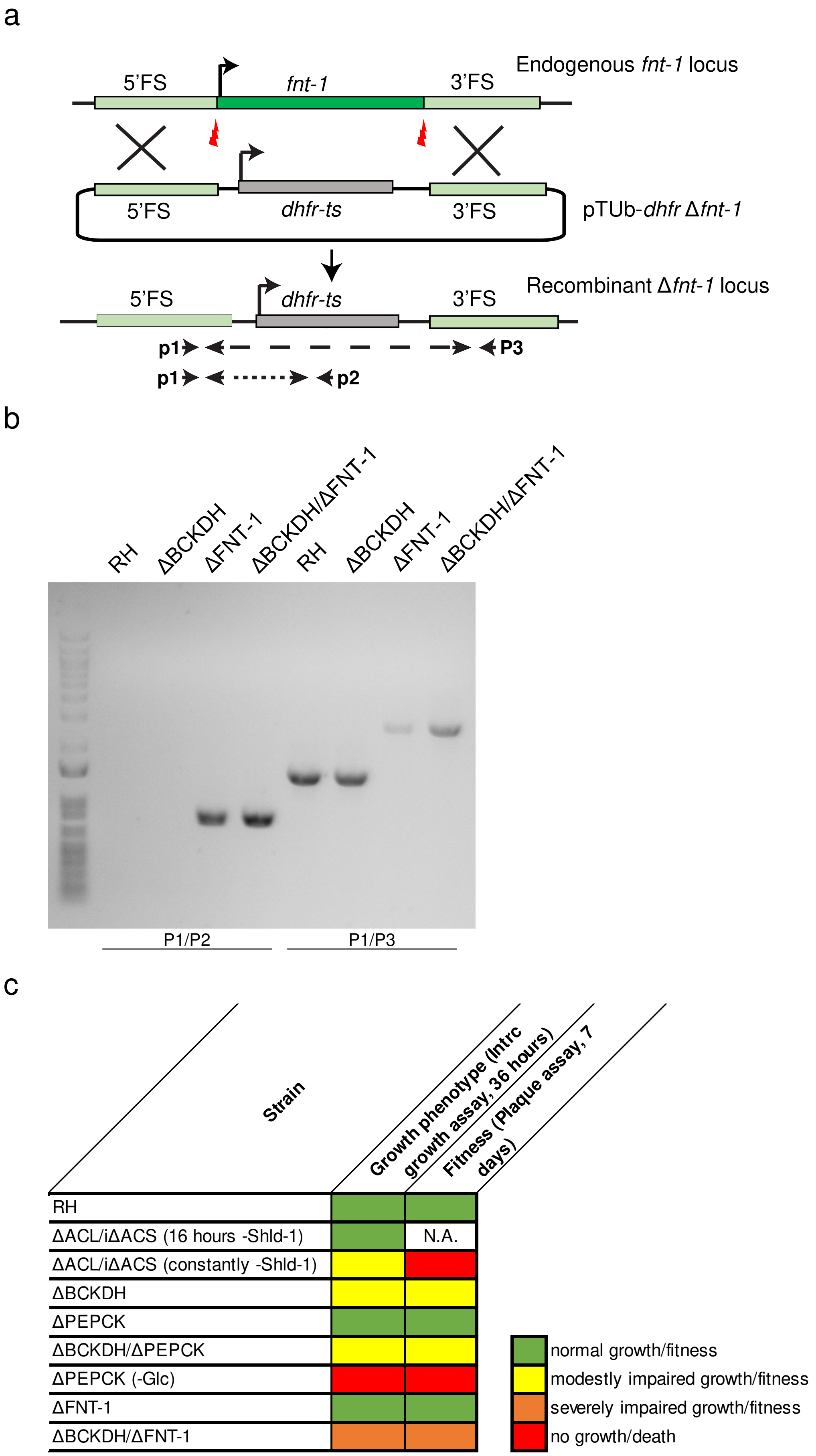

Supplement: Supplementary file 13 — Additional file 13 : Figure S6. Generation of parasites lacking BCKDH, FNT-1 or both. PDF image displaying the (a) schematic representation of the strategy to deplete the fnt-1 locus and replace it with a dhfr-ts resistance cassette. (b) PCRs were performed on genomic DNA extracted from clones and using primers listed in Additional file 14: Table S8, confirming correct integration of the constructs. (c) Table summarising the growth defect in the various strains described throughout the manuscript. Abbreviations: BCKDH: branched-chain α-keto acid dehydrogenase-complex; FS: flanking sequence; DHFR-TS: dihydrofolate reductase-thymidylate synthase; FNT: formate/nitrite transporter; PCR: polymerase chain reaction; DNA: deoxyribonucleic acid; ACL: ATP-citrate lyase; ACS: acetyl-CoA synthetase; BCKDH: branched-chain α-keto acid dehydrogenase-complex; PEPCK: phosphoenolpyruvate carboxykinase-1. [file 12915_2020_791_MOESM13_ESM.pdf]
